# Potential Waste‐to‐Wealth Strategy for Combining Medical Surgical Gloves‐Derived Pyrolysis Oil with Pentanol and Nanoparticles: Experimental Analysis on Engine Behaviors

**DOI:** 10.1002/gch2.202500031

**Published:** 2025-06-28

**Authors:** T Sathish, Ümit Ağbulut, Jayant Giri, Moaz Al‐lehaibi, Ahmad O. Hourani, Anh Tuan Hoang

**Affiliations:** ^1^ Department of Mechanical Engineering Saveetha School of Engineering SIMATS Chennai Tamil Nadu 602105 India; ^2^ Department of Mechanical Engineering Faculty of Mechanical Engineering Yildiz Technical University Istanbul 34349 Türkiye; ^3^ Department of Technical Sciences Western Caspian University Baku AZ1001 Azerbaijan; ^4^ Department of Mechanical Engineering Yeshwantrao Chavan College of Engineering Nagpur 441110 India; ^5^ Mechanical Engineering Department College of Engineering and Architecture Umm Al‐Qura University P.O. Box 5555 Makkah 24382 Saudi Arabia; ^6^ Hourani Center for Applied Scientific Research Al‐Ahliyya Amman University Amman 19328 Jordan; ^7^ Faculty of Engineering Dong Nai Technology University Bien Hoa City 76000 Vietnam; ^8^ Graduate School of Energy and Environment Korea University 145 Anam‐ro, Seongbuk‐gu Seoul 02841 South Korea

**Keywords:** alternative fuel, engine behavior, medical waste, nano‐fuel, nanoparticles, waste‐to‐fuel strategy

## Abstract

In this work, the applicability of surgical glove waste pyrolysis oil (SGWPO) combined with pentanol and nano‐additives (silicon dioxide (SiO_2_) and nano‐graphene) as a substitute fuel for a 5.2 kW‐diesel engine is experimentally conducted, aiming to analyze the test engine's performance and emission characteristics. Neat SGWPO could reach 8.6% less BTE, 32.24%, 18.27%, 36.17%, and 9.72% higher NOx, smoke, CO, and HC emissions than diesel at full load. However, blending SGWPO with 25% pentanol by volume and 100 ppm of SiO_2_ nanoparticles or 100 ppm of nanographene could improve the engine results. The obtained results of the nanographene‐included SGWPO/pentanol blend are lower than SiO_2_‐included SGWPO/pentanol blend compared to neat SGWPO. Indeed, the nano‐SiO_2_‐added SGWPO/pentanol blend demonstrated 9.72% higher BTE and 36.81%, 29.1%, 33.06%, and 25.67% reduced NOx, smoke, CO, and hydrocarbon emissions, respectively, compared to neat SGWPO. Therefore, nano‐SiO_2_‐included SGWPO/pentanol is recommended as an alternative fuel for diesel engine operation without any modifications. The recommended nanofuel blend usage in engines simultaneously supports much higher engine performance along with reduced emissions, showing the dual benefits of the waste‐to‐wealth strategy of surgical glove waste.

## Graphical abstract

This study evaluates surgical glove waste pyrolysis oil blended with pentanol and nano‐additives as an alternative fuel for diesel engines. The nano‐SiO₂ blend improved engine performance by 9.72% and significantly reduced emissions, offering a sustainable, waste‐to‐wealth solution for cleaner energy without engine modifications.

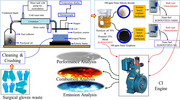

## 1 Introduction

In the world, the medical industry never gets down because of the increased population and medical needs of humans and other living things. Developing medical facilities on one side solves the health issues of the people, but on the other side, it creates pollution to the environment through their waste.^[^ ^1^ ^]^ Still, different medical wastes are there in the environment as a challenge, even though the availability of different recyclable and disposable materials, and disposal methods also support their waste influence in the environment.^[^ ^2^ ^]^ There are different types of medical waste produced every day,^[^ ^3^, ^4^ ^]^ and they are both biodegradable and non‐biodegradable.^[^ ^5^ ^]^ Among them, surgical gloves are a major plastic waste produced in every hospital. Surgical gloves are not only used by surgeons in surgery, but they are also used by doctors or medical personnel and nonmedical personnel who deal with patients for their safety.^[^ ^6^ ^]^ It is produced from hospitals, health care centers, medical clinics, research and development labs, and households.^[^ ^7^ ^]^ Normally, it is a single‐use product only in recent days, it has thus become a waste to the environment. Up to date, there has been no use in their decomposing other than waste removal.

Pyrolysis is one of the techniques to convert the medical or non‐medical plastic waste^[^ ^8^, ^9^ ^]^ into usable fuel products such as fuel for the compression ignition (CI) engine.^[^ ^10^ ^]^ In order to improve the pyrolysis performance, the plastic wastes could be co‐pyrolyzed with biomass, microalgae, or with the presence of catalysts.^[^ ^11^, ^12^ ^]^ As a result, the co‐pyrolysis process of waste nitrile gloves (25%) and neem seeds (75%) could produce bio‐oil with a heating value of 34.15 MJ kg^−1^, carbon content of 74%, and oxygen content of 13.18% in total weight.^[^ ^13^ ^]^ However, the pyrolysis oil composition and its properties depend mainly on the pyrolysis techniques. The various methods available for converting glove waste into pyrolysis oil are briefly discussed in **Table** **1**.^[^ ^14^ ^]^

**Table 1:** Feasible methods to convert glove waste into pyrolysis oil.

Method	Description	Process	Advantages	Disadvantages
Pyrolysis	Thermal decomposition of glove waste in the absence of oxygen.	Waste is shredded and fed into a pyrolysis reactor. Heated to high temperatures (300–600 °C). Produces pyrolysis oil, gas, and char.	High oil yield. Can process mixed and contaminated gloves.	Requires precise temperature control. Initial capital investment is high.
Catalytic Pyrolysis	Similar to pyrolysis, catalysts are added to lower the reaction temperature and enhance oil quality.	Zeolites, silica‐alumina, or metal oxides are the catalysts used	Improves oil quality (higher calorific value). Reduces energy consumption.	Catalyst regeneration and cost. Requires specialized equipment.
Hydrothermal Liquefaction	Converts glove waste into oil using high‐pressure water at moderate temperatures (250–350 °C).	Waste is mixed with water and heated under pressure. Produces bio‐oil, gas, and residual solids.	Suitable for wet waste. Produces fewer unwanted byproducts.	Requires high‐pressure systems. More effective with certain polymer types.
Gasification (with Oil Recovery)	Partial oxidation of glove waste at high temperatures (700–1200 °C) to produce syngas, which can be further processed into liquid fuels.	Gloves are converted into syngas (CO and H₂). Fischer–Tropsch or other processes convert syngas into synthetic oil.	Versatile end products (oil, gas, char). Effective waste‐to‐energy conversion.	Energy‐intensive. Complex equipment and process.
Thermal Cracking	Direct heating of gloves to break polymer chains into smaller hydrocarbon fractions.	Similar to pyrolysis but at higher temperatures. Produces oil, gas, and some solid residues.	Simple process. No need for complex catalysts.	Higher energy requirement. Oil quality may need refinement.
Solvent Extraction and Depolymerization	Dissolving glove polymers in a solvent to extract hydrocarbons or break them into oil‐like products.	Gloves are treated with specific solvents. Depolymerization agents help break down polymers.	Precise control over the product. Targeted conversion of specific glove types.	Expensive solvents. Solvent recovery is critical for cost‐efficiency.
Microwave‐Assisted Pyrolysis	Uses microwaves to heat glove waste uniformly, accelerating pyrolysis.	Microwave energy heats the material in an oxygen‐free environment. Produces oil, gas, and char.	Faster processing. Energy‐efficient compared to traditional pyrolysis.	Specialized reactors required. Limited large‐scale implementation.
Plasma Pyrolysis	Uses plasma (ionized gas) to achieve high temperatures to break down waste.	High‐temperature plasma decomposes polymers into oil, gas, and char.	Very high efficiency. Can handle hazardous waste.	Extremely high energy input. High operational costs.

For pyrolysis oil applications, plastic waste‐based bio‐oil could be used as a potential alternative fuel to diesel engines.^[^ ^15^, ^16^ ^]^ Indeed, 10% and 30% of bio‐oil blended with diesel fuel could be fueled to a CI engine at various loads without any modifications, showing that the highest combustion efficiency of 58% was obtained for the fuel‐air equivalence ratio of 0.91. In addition, pollutant emissions for bio‐oil/diesel blends were found to be lower than those of diesel fuel, although NOx emissions increased.^[^ ^17^ ^]^ More importantly, it was found that 100% pyrolysis oil could be used for a CI engine in the case of combining with hydrogen. In a study of Muthukumar et al.,^[^ ^18^ ^]^ they employed plastic waste‐derived bio‐oil for the 5.2‐kW CI engine with the 3–12 lpm of different hydrogen flow rates in the inlet manifold as pre‐mixing. They reported that the highest flow rate of the hydrogen‐induced condition has 16% higher BTE, 35% less smoke, 30% less unburnt hydrocarbon (HC), and 50% and 71% less carbon dioxide (CO_2_) and carbon monoxide (CO) emissions than diesel fuel. In addition to bio‐oil, alcohols can also be produced mostly by environment‐friendly techniques. Different alcohols can be used in the engine fuel operation by different techniques. Among alcohols used for engines, pentanol is one of the higher alcohols with a heating value of 35 MJ kg^−1^. It has a latent heat of evaporation of 308 kJ kg^−1^, which is higher than diesel. This alcohol produces better mixing while blending with the fuel and increases the vaporization quality during combustion in the CI engine.^[^ ^19^ ^]^ As reported in the literature, pentanol (10–30%) was blended with Jatropha oil biodiesel (90–70%) for engine application. This blending increases the heating value and reduces the viscosity of biodiesel, resulting in an increase the in‐cylinder pressure and heat release rate, and a reduction in ignition delay than diesel fuel. Resultantly, biodiesel/pentanol blend produced higher BTE (8.62%), and lower HC and CO emissions than diesel fuel, although nitrous oxide (NOx) emission from biodiesel/pentanol blend was also higher than that from diesel fuel.^[^ ^20^ ^]^

Being fuel additives, nanomaterials are the most influential creators of alternative fuels, used in engine characteristics, in which the nanoparticles are added as an additive to the fuel.^[^ ^21^ ^]^ In the literature, there have been a variety of nanoparticles used for the CI engine, including metal and non‐metal nanoparticles or carbon‐based nanoparticles. It was demonstrated that adding nanoparticles could create a catalytic reaction, an exothermic reaction, heat sink capacity, engagement in the reactivity, and micro explosions because of the dispersion and better atomization. All of these lead to improved performance and combustion, which reduces the emissions produced by the fuel.^[^ ^22^, ^23^ ^]^ Among the existing nanoparticles, silicon dioxide (SiO_2_) nanoparticles could be used to create the nano fuel with biodiesel. SiO_2_ nanoparticles were found to improve ignition and combustion duration.^[^ ^24^ ^]^ In a study of Tiwari et al.,^[^ ^25^ ^]^ 25–100 ppm of SiO_2_ nanoparticles were added to B20 (20% microalgae biodiesel + 80% diesel fuel) blend to run a CI engine under various loads. They found that the addition of SiO_2_ nanoparticles could improve brake thermal efficiency by 1.6–4.8% and brake‐specific fuel consumption by 8.55–15.33%. Moreover, SiO_2_ nanoparticles‐included B20 could reduce pollutant emissions such as ↓1.8–9% CO_2_, ↓6.2–21.4% smoke opacity, and ↓19–34% NOx. Along with SiO_2_, graphene nanoparticles are seen to have a high thermal conductivity and thermal diffusivity, showing that graphene has great potential to improve engine performance and mitigate emissions.^[^ ^26^ ^]^ Indeed, the biodiesel/diesel blend with graphene nanoparticles produced a 10% higher BTE, 5% less NOx emission, and 11% higher CO emission than diesel fuel, while CO_2_ and smoke opacity are equal to those of diesel fuel.^[^ ^27^ ^]^

From this literature, it could be realized that the use of medical surgical gloves‐derived pyrolysis oil with pentanol, and nanoparticles for diesel engines could be a promising solution to target two core goals: the waste‐to‐energy path and diversifying the alternative fuels to reduce the dependence on fossil fuels. Therefore, the novelty and necessity of this current work could be presented as follows:

1. In the context of turning medical waste, especially surgical gloves, into a sustainable fuel source, the expanding problem of managing such waste has not received enough attention.
2. Pyrolysis has been studied for other plastic wastes, but it has not been thoroughly studied for use in surgical glove waste for CI engine fuel in conjunction with performance‐enhancing additives like pentanol and nanoparticles.
3. Furthermore, little is known about how combining SGWPO with cutting‐edge nano‐additives affects engine performance and their emission.
4. The use of surgical glove waste to create pyrolysis oil, which is then combined with pentanol and improved with nanographene and nano‐SiO_2_ additives, makes this study unique.
5. Using nano‐additives to systematically improve the performance and emissions of SGWPO fuel exemplifies a novel approach.
6. An inventive step in the development of alternative fuels and waste management is marked by the study's demonstration of nano‐SiO_2_’s superior performance, which includes higher brake thermal efficiency and a significant reduction in NOx, smoke, CO, and HC emissions.

In this investigation, bio‐oil is derived from the used surgical gloves by pyrolysis, in which bio‐oil is blended with pentanol in a 3:1 ratio by volume to improve the quality of the fuel. After that, SiO_2_ nanoparticles (100 ppm) and graphene nanoparticles (100 ppm) were added separately in the blend before utilizing it for the 5.2 kW CI engine. The primary purpose of this study is to determine whether surgical glove waste pyrolysis oil (SGWPO) can be used as a substitute fuel for a CI engine and to improve its performance and emission characteristics by adding pentanol and nano‐additives. Enhancing the environmental sustainability of SGWPO as a fuel and suggesting it as a viable alternative to traditional diesel in CI engines are the objectives of the study.

## 2 Materials and Methods

**Figure 1:**
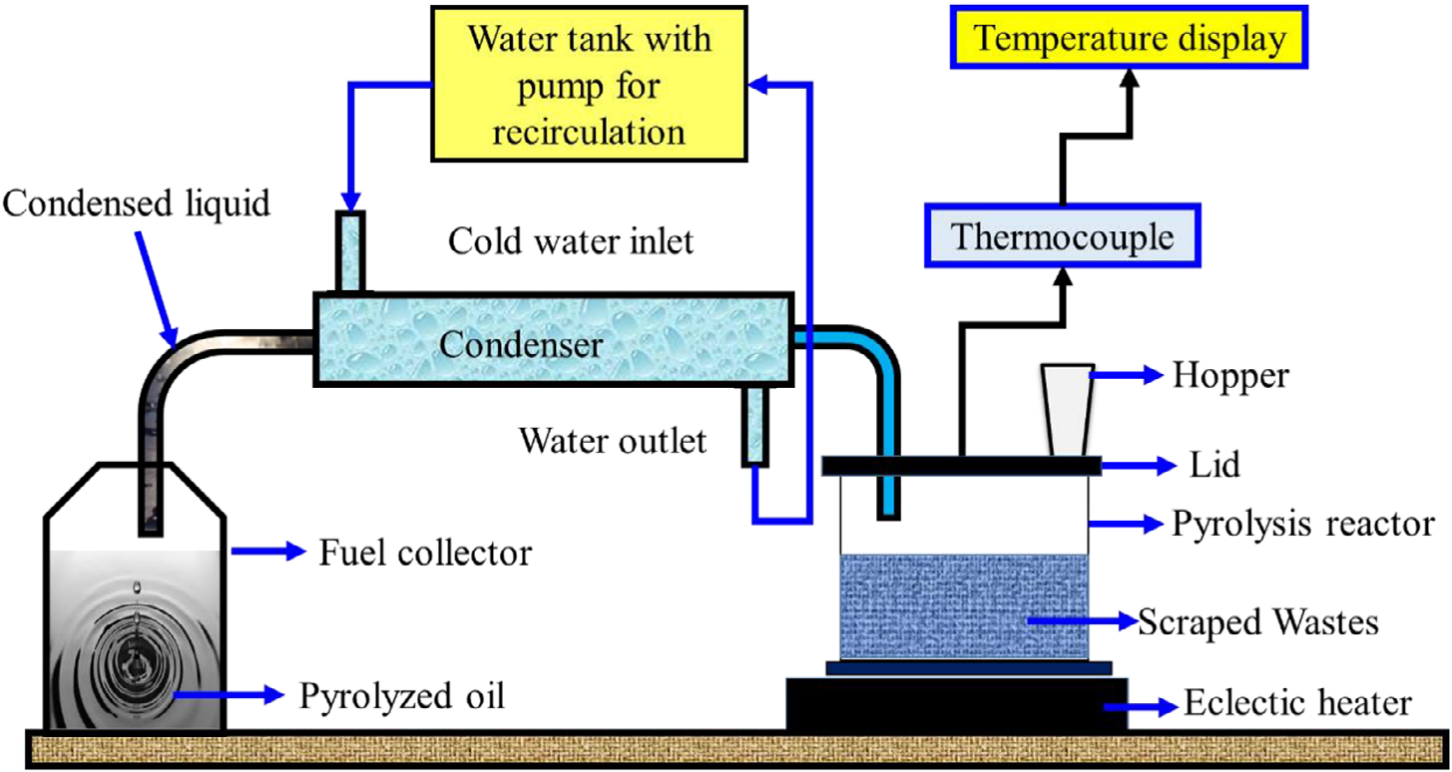
Pyrolysis of the surgical glove wastes.

This study used a bench‐scale batch pyrolysis reactor to convert surgical glove wastes into fuel. This reactor can process up to 1 kg of different plastic waste. It is made of stainless steel with glass wool insulation. With a heating rate of 10 °C min^−1^, it functions at temperatures between 450 and 500 °C, guaranteeing exact control over the pyrolysis process. To maximize fuel yield, the system allows for catalyst integration (e.g., zeolite, ZSM‐5) with a feed‐to‐catalyst ratio of 10:1. About 90 min is the retention period for products like char and pyrolysis oil. The schematic process flow is illustrated in **Figure** **1**.

Most of the medical wastes are disposed of by the incineration technique. However, their higher investment non‐incineration technique is preferred to dispose of the medical waste.^[^ ^28^ ^]^ The surgical gloves wastes are collected in various private health care hospitals and dental hospitals. The collected wastes need to be cleaned by sterilization due to their contagiousness. The steam sterilization is created with 150 °C of steam to clean the surgical gloves waste for an hour. By this steam sterilization, infection of pathogenic bacteria is destroyed.^[^ ^29^ ^]^ Then the cleaned surgical glove wastes are crushed into small particles. These are poured via a hopper into the closed pyrolysis reactor, which is heated by the electrical heater.

In the pyrolysis process, the temperature is maintained at 480 °C to produce the waste glove vapor. Then it is passed into the condenser, where the vapor condenses and converts to liquid (oil) by continuous water‐cooling. The condensed liquid is collected in the fuel collector, which is mentioned as Surgical Gloves Waste pyrolysis oil (SGWPO). The complete arrangement is shown in Figure 1. Because of its compatibility with SGWPO, its capacity to reduce viscosity, and its oxygenated structure, which improves combustion, pentanol was chosen. From the pieces of literature, 25% butanol blending provided the improved results with the alternate fuel blending from 10% to 25% of blending.^[^ ^19^, ^20^ ^]^ Because of their catalytic qualities, ability to reduce emissions, and suitability for engines without clogging or depositing, SiO_2_ nanoparticles were selected.^[^ ^30^ ^]^ Because of its dual function of increasing lubrication for engine protection and lowering emissions and calorific value, nanographene was added.^[^ ^31^ ^]^ Hence, the SiO_2_ nanoparticles optimize combustion efficiency and drastically cut harmful emissions, while pentanol enhances the fuel's overall combustion profile and makes it more engine‐compatible. In addition to improving fuel efficiency, nanographene offers further advantages in lowering emissions. Based on experimental benchmarks and studies that balance cost, practicality for real‐world applications, and effectiveness, 100 ppm for the nanoparticles and 25% by volume for pentanol were chosen. With the help of this refined mixture, surgical glove waste pyrolysis oil is converted into a practical, environmentally responsible alternative fuel that promotes sustainability and effective engine performance.

**Figure 2:**
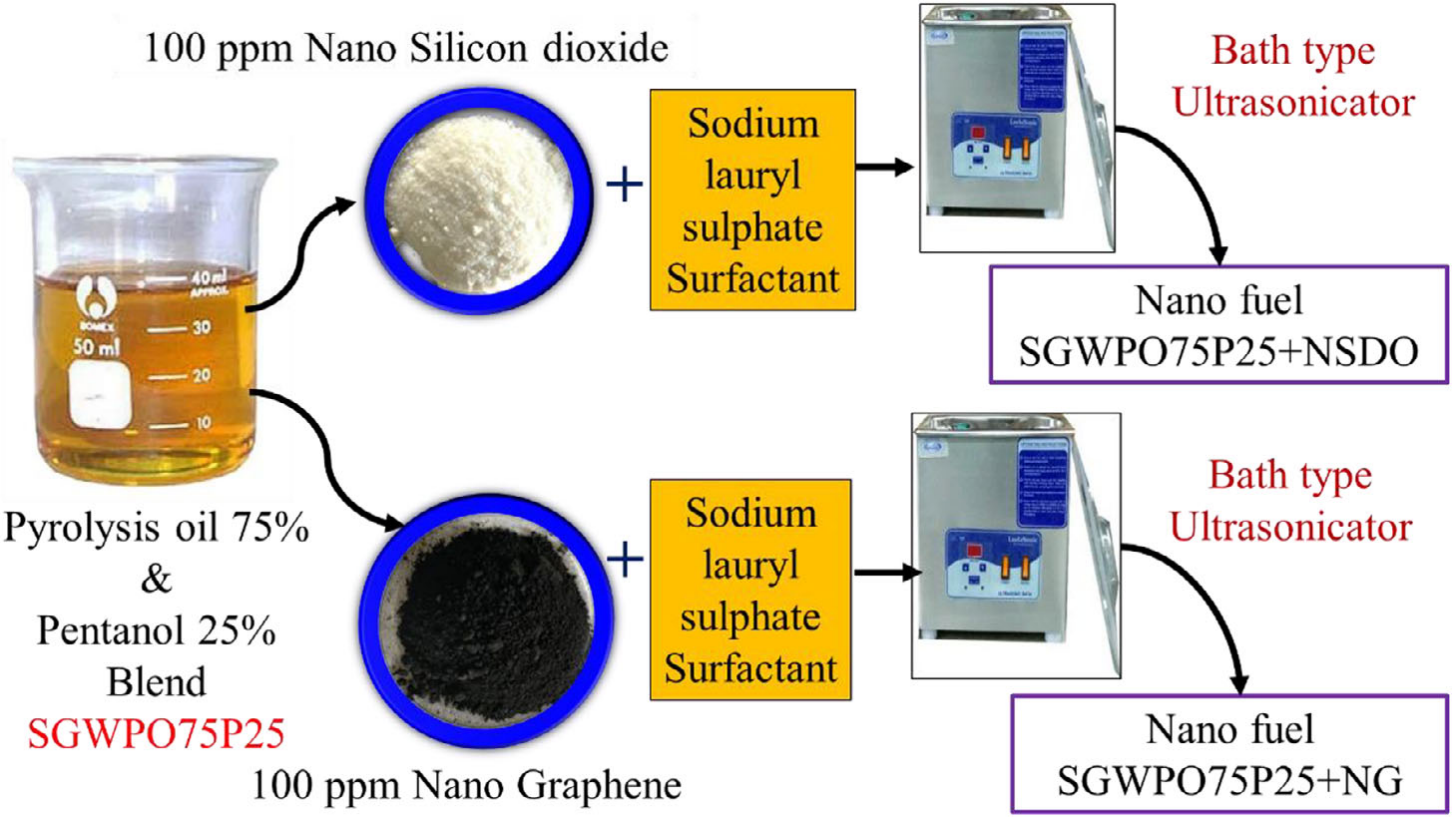
SGWPO75P25+NSDO and SGWPO75P25+NG preparation.

The basic required properties of SGWPO are measured and tabulated in **Table** **2**. The viscosity and density of the SGWPO are higher than diesel, and the calorific value is lower than diesel. In order to improve these properties, pentanol is blended with the SGWPO at a 1:3 ratio by volume. 75% of SGWPO and 25% of pentanol are used to produce the improved SGWPO blend. This is mentioned as SGWPO75P25. To improve the fuel quality, addition of nano SiO_2_ (99.8% purity, white, 30–50 nm, RCK chemicals – Chennai) and nanographene (99.5% purity, Black, 30–50 nm, RCK chemicals – Chennai) is created separately 100 ppm per litter of blend with the surfactant of Sodium lauryl sulphate (5 mL/lit) in a bath type ultra sonicator (20 kW, 20–100 kHz, 5 lit capacity) at 50 kHz frequency for 50 min duration. This nano‐fuel preparation flow is mentioned in **Figure** **2**.

**Table 2:** SGWPO, blend, nano‐fuel properties comparison with diesel.

Properties	Unit	Diesel	SGWPO	Pentanol – P	SGWPO75P25	SGWPO75P25+NSDO	SGWPO75P25+NG	ASTM standard
Viscosity	cSt	2.66	3.2	2.9	3.125	3.12	3.12	D 792
Flash point	°C	74	89	56	80	78	79	D 93
Density	kg m^−3^	827	853	816	838	838	838	D 4052
Cetane number	–	51	45	20	48	49	49	D 8183
Calorific value	MJ kg^−1^	42.24	39.25	35.56	40.25	41.3	41.05	D 5868

Ultimate analysis						
Content	Carbon [wt%]	Hydrogen [wt%]	Nitrogen [wt%]	Oxygen [wt%]	Sulfur [wt%]	Carbon residue [wt%]
Diesel	88.4	11.09	0.01	0.149	0.03	0.009
SGWPO	86.3	13.02	0.15	0.2	0.009	0.18

The nano‐fuel of nano SiO_2_ used blend is mentioned as SGWPO75P25+NSDO and the nanographene used blend is mentioned as SGWPO75P25+NG. These prepared nano‐fuels (100 ml) are kept separately in a test tube for 10 days, there is no sedimentation of the nano particles added, showing that these nano‐fuels have good stability.

## 3 Experimental Setup

**Figure 3:**
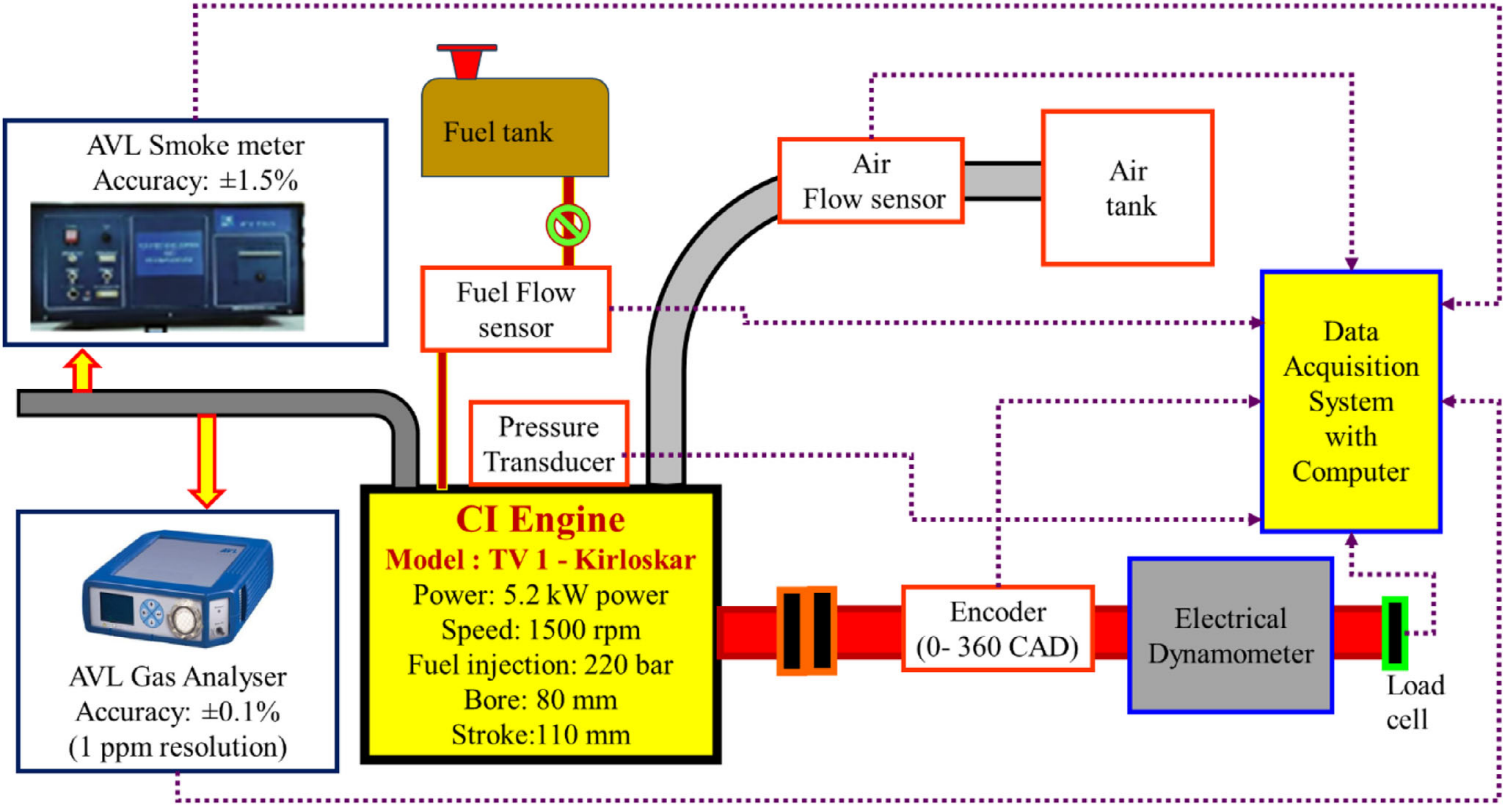
CI engine experimental setup.

The investigational engine arrangement is mentioned in **Figure** **3**. The single‐cylinder CI engine is connected to the electrical dynamometer with a load cell for the purpose of applying a load. The crank angle encoder and pressure transducer are connected to measure the pressure variation inside the cylinder and the position of the crank. The flow of air supplied to the intake manifold and the flow of fuel supplied to the engine were obtained by using separate flow meters connected in their flow path.

The produced smoke in the exhaust is identified with the AVL smoke meter, and the produced gaseous emission in the exhaust gases is measured with the gas analyzer. All these measuring and sensing elements are connected with the data acquisition system as per Figure 3, and the data is stored in the computer with the help of the Engine software. The specifications of the engine and the equipment used, along with their uncertainty, are mentioned in **Table** **3**.

**Table 3:** Specification of the list of equipment used in the experimental arrangement with individual and total uncertainty.

5.2 kW power CI Engine									
Speed	Compression ratio		Model		Make	Cooling		
1500 rpm	17.5		TV1	Kirloskar		Water		
**Cylinder Dimension**				**Bore**		**0.0875 m**			
				**Stroke**		**0.11 m**			
**Injection – Direct**				Pressure		200 bar			
				Timing		23° TDC			
**Gas analyser (AVL)**	**CO**		**UHC**	**NOx**	**Smoke meter (AVL)**		
Accuracy	0.001%		1 ppm	2 ppm	0.02%		
Uncertainty (U)	±0.5%		±1.3%	±0.6%	± 1.0%		
Others	**BP**		**BTE**	**Temperature – T**			
Accuracy	0.001 kW		0.01%	0.05 °C			
Uncertainty (U)	± 1.0%		± 0.5%	± 1.3%			

Content	Trail 1	Trail 2	Trail 3	µ	σ	%U_0.95_	IE	%IU
CO	15.13	15.14	15.15	15.14	0.010	0.13	0.5	0.5
UHC	0.96	0.95	0.96	0.96	0.006	1.21	0.5	1.3
Nox	11.24	11.25	11.21	11.23	0.021	0.37	0.5	0.6
T	96	95	96	95.67	0.577	1.21	0.5	1.3
BP	5.18	5.22	5.19	5.20	0.021	0.80	0.6	1.0
BTE	30.59	30.51	30.65	30.58	0.070	0.46	0.01	0.5
Smoke	188.2	188.3	188.4	188.30	0.100	0.11	1.01	1.0
Total Uncertainty (TU)								± 2.51

The means of trials found by Equation (1), the standard deviation found by Equation (2), the uncertainty of the data found with Equation (3), the individual uncertainty found by Equation (4) with instrumental error, and the total uncertainty are measured with the individual uncertainties as given by Equation (5).^[^ ^32^ ^]^

(1) $$Mean\;\left(\mu \right)=\frac{\sum Trails_{indivual}}{Number\;of\;trails}$$

(2) $$Standard\;deviation\;\left(\sigma \right)=\sqrt[{2}]{\frac{\sum {\left(Individual\;trails-\;\mu \right)}^{2}}{Number\;of\;count}}$$

(3) $$Uncertainty\;of\;data=\%U_{0.95}=\frac{2X\sigma X100}{\mu }$$

(4) $$Individual\;Uncertainty=\%IU=\sqrt[{2}]{\%{U_{0.95}}^{2}+IE^{2}}$$

(5) $$Total\;Uncertainty=TU=\sqrt[{2}]{\begin{array}{c} {\left(U_{BTE}\right)}^{2}+{\left(U_{speed}\right)}^{2}+{\left(U_{temperature}\right)}^{2}\\ +{\left(U_{time}\right)}^{2}+{\left(U_{smoke}\right)}^{2}+{\left(U_{NOx}\right)}^{2}\\ +{\left(U_{CO2}\right)}^{2}+{\left(U_{UHC}\right)}^{2}+{\left(U_{CO}\right)}^{2}+{\left(U_{BP}\right)}^{2} \end{array}}$$

**Figure 4:**
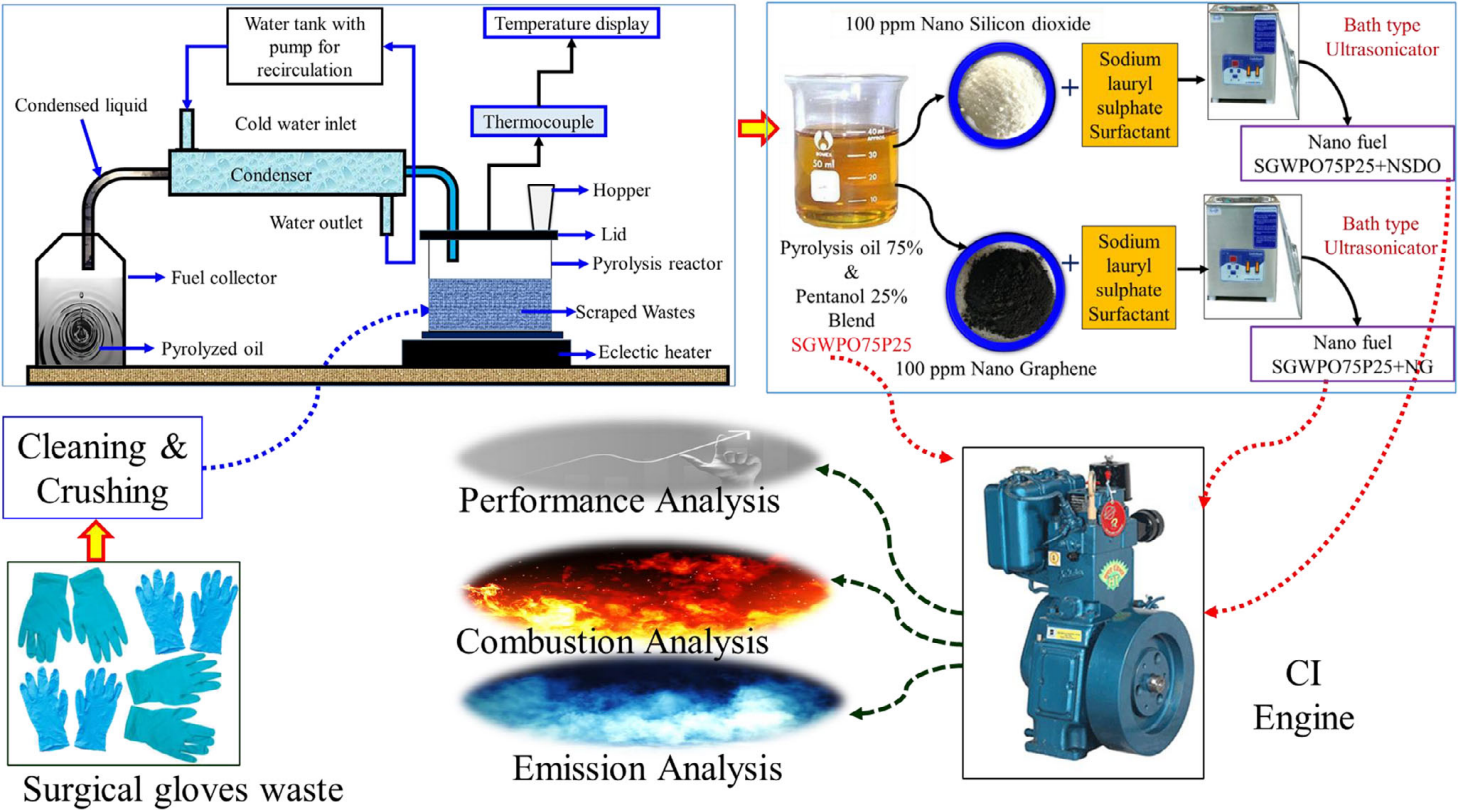
Graphical representation of this investigation.

The complete flow of this experimental investigation is mentioned in **Figure** **4**. The test fuels and their proportions of the fuel, pentanol, and nanoparticles are mentioned in **Table** **4**. Each fuel is used to run the engine at zero to full load conditions. There are 4 times to replicate the experiment to obtain the mean value. The brake‐specific fuel consumption (BSFC), Brake thermal efficiency (BTE) and Heat release rate (HRR) were calculated by Equations (6)–(8).^[^ ^18^ ^]^

**Table 4:** Test trial details of the investigation.

Test name	Diesel	SGWPO	Pentanol	NSDO ‐Nano silicon dioxide	NG ‐Nanographene
Diesel	100%	0%	0	0	0
SGWPO	0%	100%	0%	0	0
SGWPO75P25	0%	75%	25%	0	0
SGWPO75P25+NSDO	0%	75%	25%	100 ppm	0
SGWPO75P25+NG	0%	75%	25%	0	100 ppm

BSFC and BTE values are calculated as in Equations (6) and (7).

(6) $$BSFC=\frac{{\dot{m}}_{f}\times 10^{3}}{BP}\times 3600$$

Where, ${\dot{m}}_{f}\;\mathrm{and}\;BP\;$ are the fuel mass flow rate (g/s) and brake power (kW).

(7) $$BTE=\frac{BP\times \;3600\;}{{\dot{m}}_{f}\;\times \;LHV_{f}}x100\;\left(\%\right)$$

(8) $$\begin{array}{ccc} Heat\;release\;rate\left(HRR\right)=\frac{dQ}{d\emptyset } & = & \left\{\left(\frac{\gamma }{\gamma -1}\right)P\left(\frac{dV}{d\emptyset }\right)\right\}+\\ & & \left\{\left(\frac{1}{1-\gamma }\right)V\left(\frac{dP}{d\emptyset }\right)\right\} \end{array}$$

## 4 Results and Discussion

The measured and calculated experimental data are presented as a graphical representation in the following section with explanations. Initially, the combustion of the engine with respective fuels and emission variation for the same fuels were explained. Finally, the variations in the performance were discussed with supporting quotes from the previous results by citations.

### 4.1 Blends and Nano Additives Influence the SGWPO in the Combustion Analysis

**Figure 5:**
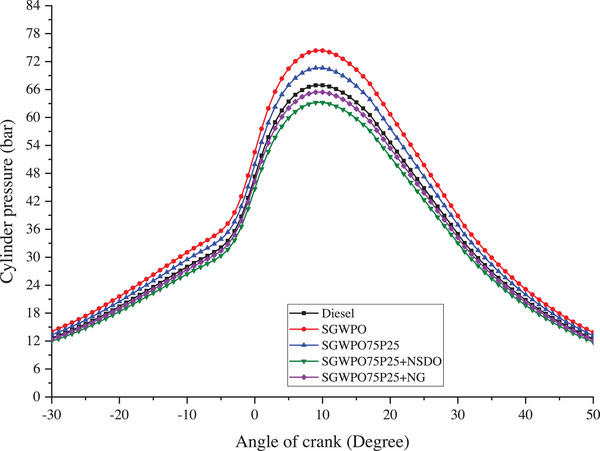
In‐cylinder pressure variation by blends and nano additives influence on the SGWPO in the combustion analysis.

The rate of heat release during combustion, which is dependent on the calorific value, fuel's volatility, and fuel atomization, affects the peak in‐cylinder pressure (ICP). In‐cylinder pressure (ICP) variation due to the blends and nano additives influence on the SGWPO in the combustion analysis is mentioned in **Figure** **5** at full load.

The highest peak pressure of 74.34 bar is produced by SGWPO, and the lowest peak pressure of 63.19 bar is obtained by SGWPO75P25+NSDO. Compared to diesel, SGWPO has an 11.11% increase in peak pressure due to the higher viscosity and density of the SGWPO, which produced poor volatility and poor vaporization while injected into the cylinder during the combustion.^[^ ^33^, ^34^ ^]^ The addition of the pentanol increases the vaporization quality of the fuel with reduced viscosity and improved calorific value, therefore, SGWPO75P25 has produced 5% less peak pressure than SGWPO.^[^ ^35^ ^]^ SGWPO75P25+NG has 2.2% and 12% less peak pressure than diesel and SGWPO, respectively. This reduction is because of the better calorific value of the nano‐fuel.^[^ ^27^, ^36^ ^]^

Similarly, SGWPO75P25+NSDO has 5.56% and 14.98% less peak pressure than the diesel and SGWPO, respectively. This reduction is because of the highest heating value of the nano fuel and increased secondary fuel atomization caused by the nano additives.^[^ ^21^, ^22^, ^23^ ^]^ Due to its higher density and viscosity than diesel, SGWPO causes delayed vaporization and poor atomization during injection. As a result, there is more fuel built up in the cylinder before combustion and a longer ignition delay. Higher peak pressures result from the rapid increase in heat release rate that occurs when combustion starts. Pentanol's reduced viscosity enhances SGWPO's spray properties and vaporization quality. As reported in literature, oxygenated fuels could enhance the fuel oxidation, thus improving combustion.^[^ ^37^ ^]^ The blend's enhanced volatility and decreased viscosity results in a more controlled and seamless combustion process. The ignition delay is decreased, and the heat release is more uniformly distributed when pentanol is added. As a result, SGWPO75P25 generates a 5% lower peak pressure than SGWPO due to a less abrupt combustion process that lessens pressure spikes.

Because of the high thermal conductivity of nanographene, it releases energy more efficiently during combustion by increasing the rate of heat transfer. Because of its lubricating qualities, the blend flows and atomizes better, reducing unburned fuel and facilitating more thorough combustion. As a result, the SGWPO75P25+NG blend has a higher calorific value and better combustion dynamics, which lowers peak pressure by 2.2% when compared to diesel and 12% when compared to SGWPO. This suggests that the energy release is more controlled and gradual, which lowers pressure peaks. As catalysts for combustion, SiO_2_ nanoparticles improve fuel‐air mixture oxidation and lessen combustion irregularities. Nanoparticles' enhanced atomization lowers the injected fuel's droplet size, improving mixing and accelerating vaporization. These enhancements to the SGWPO75P25+NSDO fuel blend result in a more consistent combustion process with higher secondary atomization.

As a result, the peak pressure is 14.98% lower than SGWPO and 5.56% lower than diesel. The peak pressure is further lowered by the slower rate of pressure rise in the cylinder caused by the higher heating value and controlled combustion. Therefore, the main findings are that using SGWPO alone as fuel in a diesel engine produced the highest peak pressure (74.34 bar) because of its limited volatility, which led to sudden combustion following delayed ignition. Because pentanol has better volatility and less viscosity than SGWPO, it was found that employing SGWPO75P25 resulted in a 5% lower peak pressure and more uniform combustion. Because of the increased calorific value and the combustion‐improving qualities of nanographene, the usage of the SGWPO75P25+NG fuel mix decreased the peak in cylinder pressure by 2.2% when compared to diesel and 12% when compared to SGWPO.

The best atomization and combustion catalysis by SiO_2_ nanoparticles resulted in the lowest peak pressure (63.19 bar), with reductions of 5.56% compared to diesel and 14.98% compared to SGWPO when using SGWPO75P25+NSDO blend. **Table** **5** demonstrates the comparative analysis of fuel blends’ performances in terms of In‐Cylinder Pressure and highlights the key factors influencing the results.

**Table 5:** Comparison of Fuel Performances at full load in terms of in‐cylinder pressure.

Fuel blend	Peak pressure [bar]	Change vs diesel [%]	Change vs SGWPO [%]	Key factors influencing ICP
Diesel	66.92	Baseline	−11.11%	Optimal vaporization, combustion quality
SGWPO	74.34	11.11%	Baseline	High viscosity and density lead to delayed combustion and peak pressure rise
SGWPO75P25	70.62	5.52%	−5.00%	Pentanol's improved volatility and oxygen content
SGWPO75P25 +NG	65.46	−2.20%	−12.00%	Nanographene enhances combustion but reduces peak pressure due to uniform heat release
SGWPO75P25 +NSDO	63.19	−5.56%	−14.98%	SiO_2_’s atomization effect and high heating value enable smoother combustion

**Figure 6:**
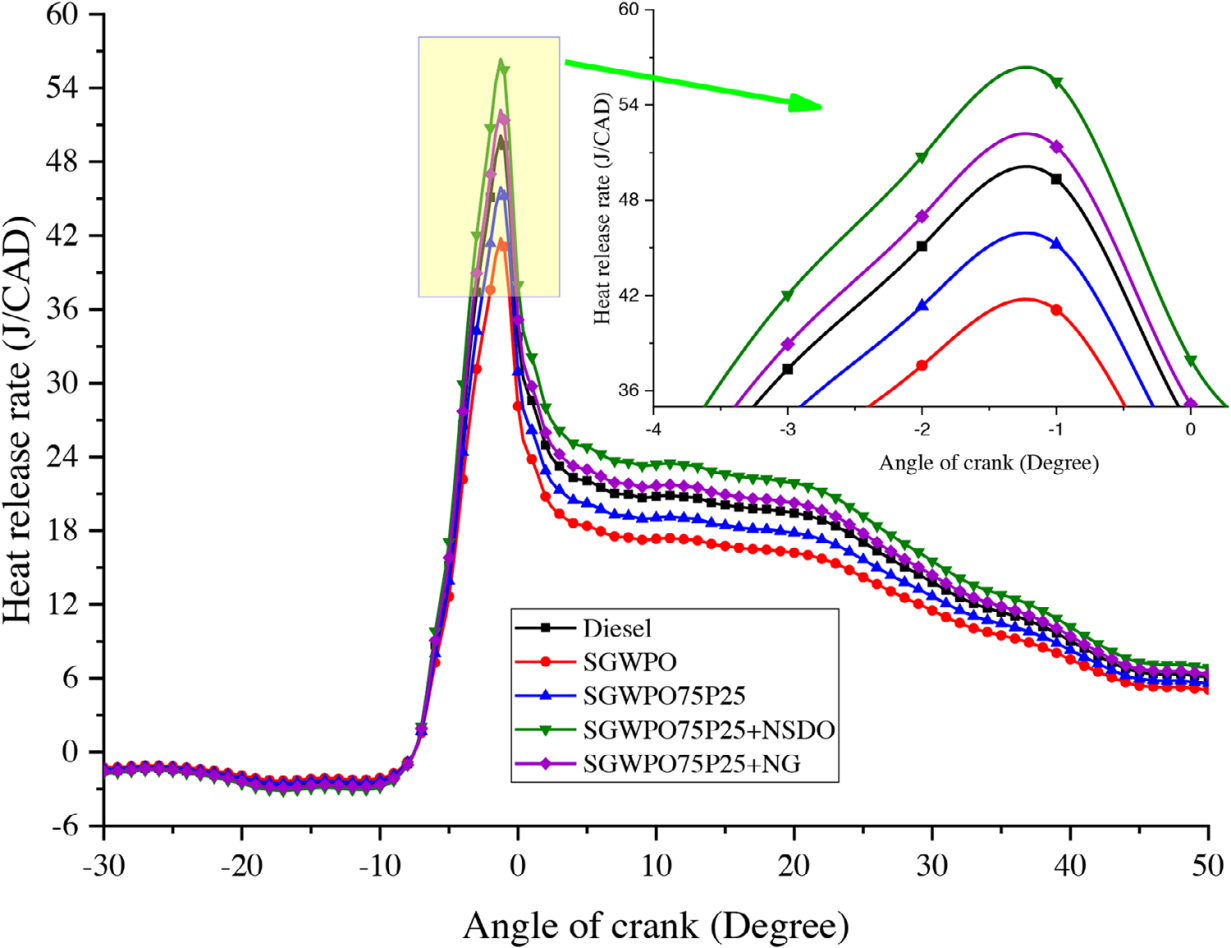
Heat release rate variation by blends and nano additives influence on the SGWPO in the combustion analysis.

The rate of energy release during combustion, which is impacted by the calorific value, vaporization, and mixing quality of the fuel, determines the Heat release rate (HRR). Maximum HRR variation by blends and nano additives influences the SGWPO in the combustion analysis, as mentioned in **Figure** **6** at full load. The lowest maximum HRR of 41.10 J/CAD is produced by SGWPO, and the highest HRR of 55.48 J/CAD is obtained by SGWPO75P25+NSDO. Compared to diesel, SGWPO has a 16.67% reduced maximum HRR due to the lower heating value of the fuel and poor vaporization created by the higher viscosity and density of SGWPO.^[^ ^8^ ^]^ However, the pentanol addition increases calorific value, improves vaporization and reduces viscosity; therefore, SGWPO75P25 has produced a 10.02% increase in maximum HRR compared to the SGWPO.^[^ ^19^, ^20^ ^]^

The increased heating value of SGWPO75P25+NG has produced 4.7% and 24.94% higher maximum HRR than diesel and SGWPO, respectively. This increase in HRR is because of the higher calorific value of the nano fuel and thermal engagement of the nanographene.^[^ ^31^, ^38^ ^]^ Similarly, SGWPO75P25+NSDO has 12.5% and 35.04% higher maximum HRR than the diesel and SGWPO, respectively. This increase is because of the higher heating value of the nano fuel, better oxidation and increased micro explosions triggered by the nanoparticles.^[^ ^30^, ^39^, ^40^ ^]^

The high density and viscosity of SGWPO hinder vaporization and atomization. Inadequate vaporization lowers combustion efficiency and delays fuel‐air mixing. SGWPO's lower calorific value restricts the amount of energy that can be released as heat. When compared to diesel, the maximum HRR is reduced by 16.67% due to incomplete combustion and slower heat release caused by poor volatility of SGWPO. SGWPO's high density and viscosity result in a less‐than‐ideal fuel‐air combination, which further restricts the rate of combustion. The presence of pentanol in the SGWPO75P25 fuel blend enhances vaporization and lowers viscosity, facilitating improved fuel atomization and mixing. Additionally, pentanol has a larger calorific value and oxygen content than SGWPO, which improves combustion efficiency. The SGWPO75P25 blend's enhanced fuel qualities lead to quicker and more thorough combustion. Since the mixture releases more energy due to higher combustion kinetics, the maximum HRR rises by 10.02% when compared to SGWPO. The SGWPO75P25+NG blend's excellent thermal conductivity of nanographene enhances heat transmission during combustion, speeding up heat release and improving ignition. The lubricating qualities of nanographene enhance spray characteristics by decreasing droplet size and boosting combustion surface area. Better atomization and a higher calorific value result in increased combustion, which raises HRR by 24.94% when compared to SGWPO and 4.7% when compared to diesel. Faster combustion is made possible by the more effective energy release made possible by nanographene. By improving fuel‐air mixing and decreasing ignition delay, SiO_2_ nanoparticles in the SGWPO75P25+NSDO blend function as combustion catalysts, improving oxidation.

Larger fuel droplets are broken up into smaller ones by the nanoparticles' induction of micro‐explosions in the fuel. This accelerates the process of heat release by increasing the contact surface area for combustion. Of all the blends, the most efficient combustion is the result of the enhanced oxidation and micro‐explosion effects. The blend's higher calorific value, improved atomization, and accelerated oxidation all work together to provide an HRR that is 35.04% higher than SGWPO and 12.5% higher than diesel. **Table** **6** demonstrates the comparative analysis of fuel blends’ performances in terms of HRR and highlights the key factors influencing the results.

**Table 6:** Comparison of fuel performances at full load in terms of heat release rate.

Fuel blend	Maximum HRR [J/CAD]	Change vs diesel [%]	Change vs SGWPO [%]	Key factors influencing HRR
Diesel	49.32	Baseline	16.67%	Optimal heating value, vaporization, and combustion
SGWPO	41.1	−16.67%	Baseline	Lower heating value, poor vaporization due to high viscosity and density
SGWPO75P25	45.21	−8.34%	10.02%	Pentanol improves calorific value, vaporization, and reduces viscosity
SGWPO75P25 +NG	51.64	4.70%	24.94%	Nanographene enhances thermal engagement and heating value
SGWPO75P25 +NSDO	55.48	12.50%	35.04%	Silicon dioxide's oxidation, micro‐explosion, and higher combustion efficiency

### 4.2 Blends and Nano Additives Influence on the SGWPO in the Emission Analysis

**Figure 7:**
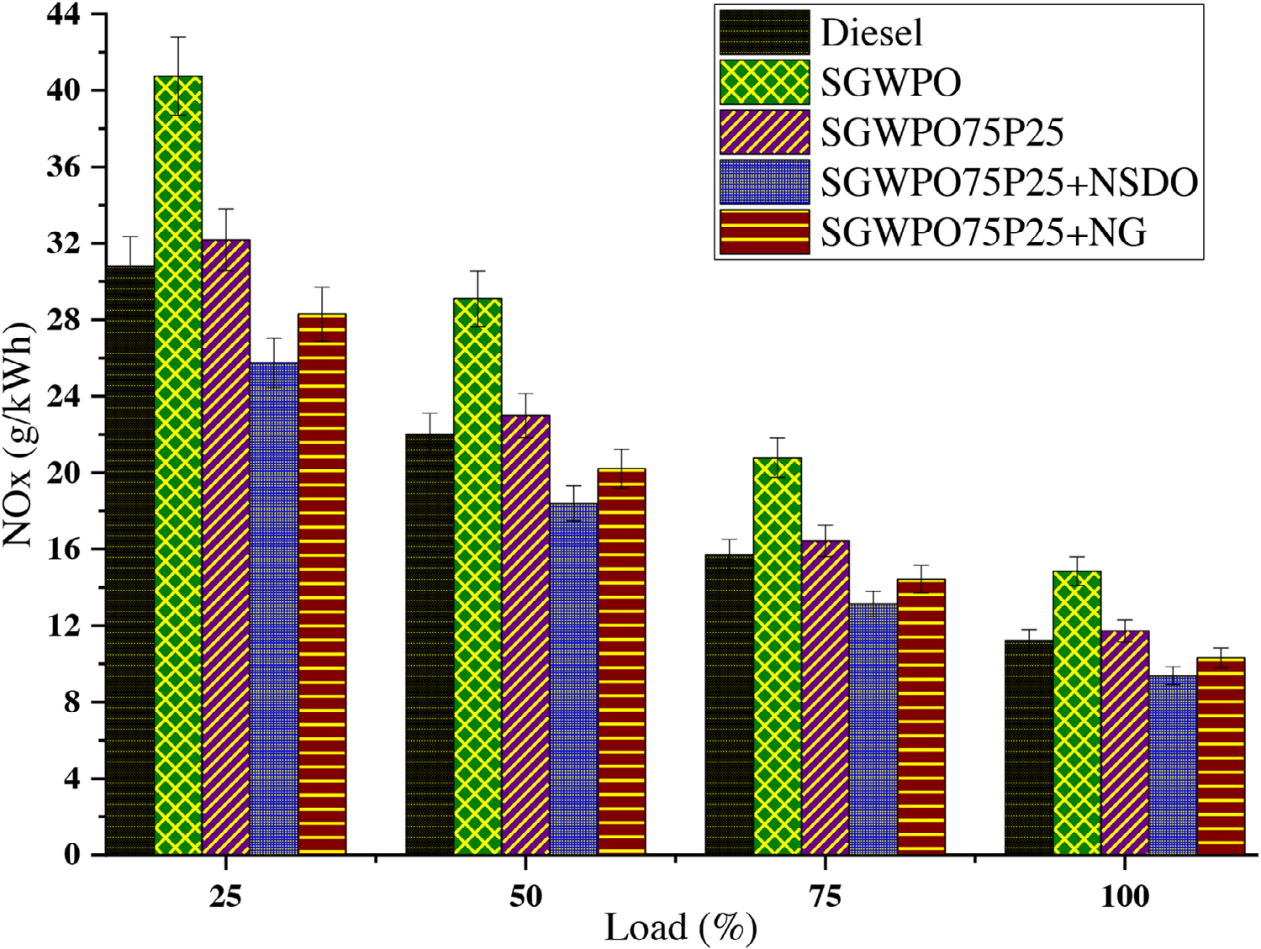
NOx emission variation by blends and nano additives influences the SGWPO in the emission analysis.

The temperature and oxygen availability inside the cylinder have a significant impact on NOx production. The process by which nitrogen and oxygen react to form NOx at high temperatures is accelerated by higher temperatures. NOx emissions variation by blends and nano additives influences the SGWPO in the combustion analysis, as mentioned in **Figure** **7** at different loads. The observed data in this section were statistically analyzed and plotted with error bars at a 95% confidence interval at ± 1 standard deviation. Compared to a lower load, the higher load has lesser NOx emissions for all the test fuels. At maximum load, the highest NOx emission of 14.85 g kWh^−1^ is produced by SGWPO, and the lowest NOx emission of 9.38 g kWh^−1^ is obtained by SGWPO75P25+NSDO. Compared to diesel, SGWPO has a 32.24% increase in NOx emission due to the increase in in‐cylinder pressure of SGWPO, which increases the temperature inside the cylinder, this promotes the higher NOx emission.^[^ ^18^, ^41^ ^]^

The pentanol addition produces better evaporation and supplies oxygen content to combustion, increasing the heating value and better vaporization, reducing the in‐cylinder temperature. The pentanol creates a cooling effect in the combustion chamber, which reduces the temperature of the in‐cylinder and simultaneously reduces 21% of NOx emissions than the SGWPO.^[^ ^20^, ^35^ ^]^ The SGWPO75P25+NG has produced 8.19% and 30.57% reduced NOx emissions compared to diesel and SGWPO, respectively. This reduction in NOx is because of both pentanol and graphene nanoparticles in the fuel. Pentanol produced better evaporation and additional oxygen content for combustion. Nanographene produces thermal engagement and heat sink quality, which reduces the cylinder temperature, which reduces the NOx emission.^[^ ^31^, ^38^ ^]^ Similarly, SGWPO75P25+NSDO has 16.44% and 36.81% less NOx emission than the diesel and SGWPO, respectively. This reduction is because of both pentanol and the nano SiO_2_ involvement in the combustion. The improved evaporation by the pentanol creates a cooling effect on the cylinder, and SiO_2_ nanoparticles produce a higher catalytic engagement, which converts the produced NO emissions into nitrogen. This reduction also creates a higher reduction in the NOx emission.^[^ ^19^, ^23^ ^]^

The increased density and viscosity of SGWPO cause delayed combustion and inadequate atomization. By raising the temperature and pressure inside the cylinder, these elements encourage the production of more NOx. Compared to diesel, SGWPO produces 32.24% more NOx emissions because of its combustion properties, which raise the in‐cylinder temperature. The cooling action of pentanol lowers the in‐cylinder temperature when using the SGWPO75P25 blend. Blends of pentanol show improved atomization and evaporation, which leads to a more consistent combustion process. Because of its cooling properties, pentanol's oxygen content enhances combustion quality without causing uncomfortably high temperatures. When compared to SGWPO, the SGWPO75P25 fuel's improved evaporation and decreased viscosity lowers peak in‐cylinder temperatures and cut NOx emissions by 21%.

The localized high‐temperature zones that cause NOx generation are stopped by the cooling action. When employing the SGWPO75P25+NG blend, the thermal conductivity and heat sink characteristics of nanographene aid in better heat dissipation within the cylinder, avoiding an excessive temperature rise. Localized high‐temperature zones are decreased by pentanol and nanographene's improved atomization and uniform burning. By serving as a thermal regulator, nanographene lowers NOx emissions by 8.19% when compared to diesel and 30.57% when compared to SGWPO. Pentanol and nanographene work together to provide controlled temperature profiles and effective combustion. By acting as a combustion catalyst and facilitating the conversion of NO into innocuous nitrogen and oxygen, SiO_2_ nanoparticles, when using the SGWPO75P25+NSDO blend efficiently lower NOx emissions. By improving fuel evaporation and producing a cooling effect, the pentanol blend lowers peak in‐cylinder temperatures. By altering the combustion chemistry, SiO_2_’s catalytic action lowers the production of heat NOx. Pentanol's cooling qualities and SiO_2_’s catalytic action work together to reduce NOx emissions by 36.81% when compared to SGWPO and 16.44% when compared to diesel. Pentanol's uniform and thorough combustion reduces high‐temperature zones, and SiO_2_ actively inhibits the formation of NOx and improves post‐combustion reduction. **Table** **7** demonstrates the comparative analysis of fuel blends’ performances in terms of NOx emissions and highlights the key factors influencing the results.

**Table 7:** Comparison of fuel performances at full load in terms of NOx emissions.

Fuel blend	NOx Emissions [g kWh^−1^]	Change vs diesel [%]	Change vs SGWPO [%]	Key factors influencing NOx emissions
Diesel	11.23	Baseline	−32.24	Moderate in‐cylinder temperature and balanced combustion characteristics
SGWPO	14.85	+32.24	Baseline	Higher in‐cylinder temperature due to increased pressure, leading to more NOx formation
SGWPO75P25	11.73	+4.45	−21.00	Cooling effect of pentanol by reducing the in‐cylinder temperature, improved vaporization
SGWPO75P25+NG	10.31	−8.19	−30.57	Pentanol improves evaporation, nanographene, provides heat sink properties, reducing cylinder temperature
SGWPO75P25+NSDO	9.38	−16.44	−36.81	Cooling effect of pentanol and catalytic engagement of SiO_2_ converting NO into nitrogen

**Figure 8:**
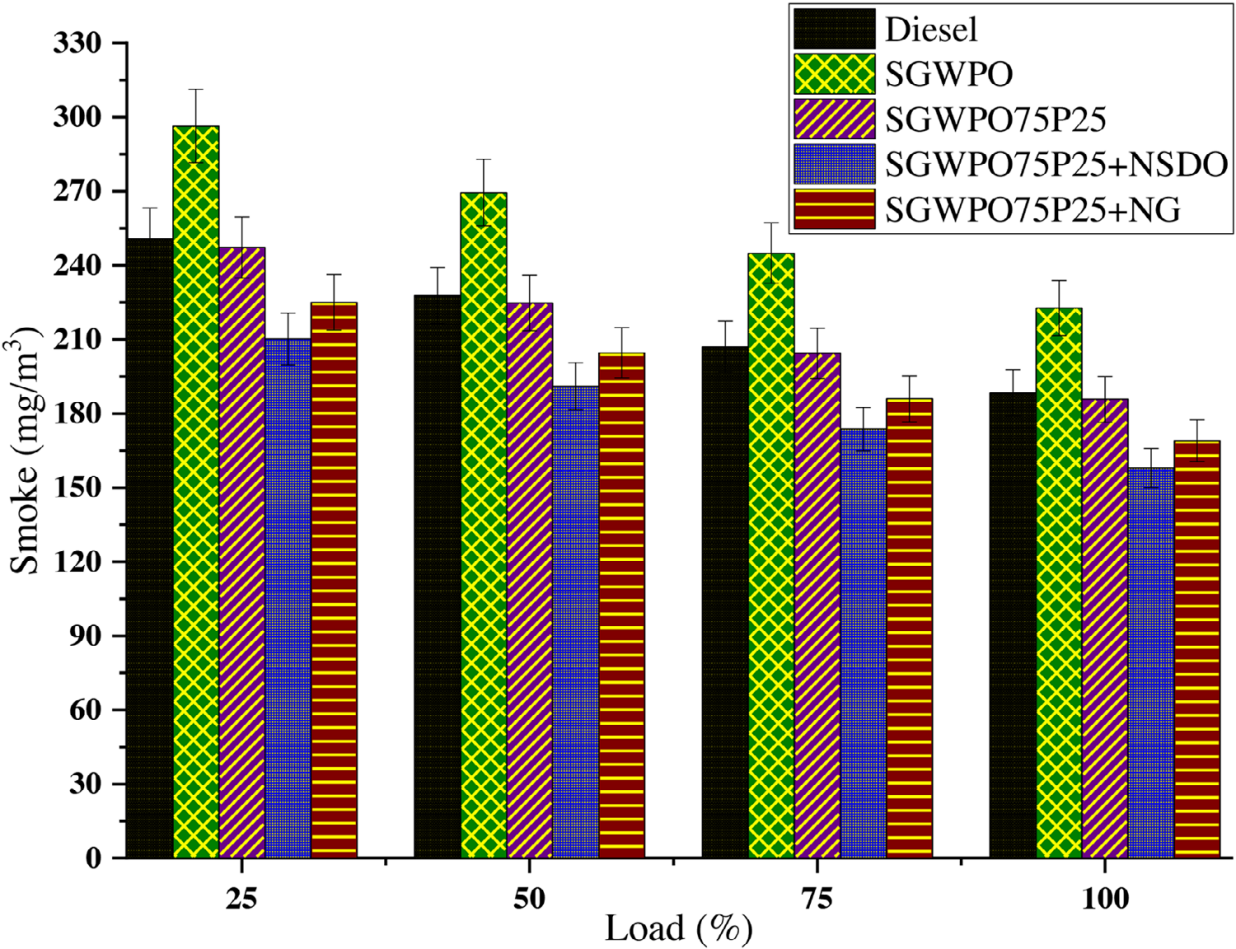
Smoke emission variation by blends and nano additives influences on the SGWPO in the emission analysis.

Incomplete combustion produces carbon soot particles, which in turn cause smoke emissions. Smoke emissions variation due to blends and nano additives influence the SGWPO in the combustion analysis is mentioned in **Figure** **8** at different loads. Compared to a lower load, the higher load has lesser smoke emission for all the test fuels. At maximum load, the highest smoke emission of 222.7 m^3^ kg^−1^ is produced by SGWPO, and the lowest smoke emission of 157.9 m^3^ kg^−1^ is obtained by SGWPO75P25+NSDO. Compared to diesel, SGWPO has an 18.27% increase in smoke emissions due to high viscosity and poor volatility. Delayed combustion with higher viscosity and poor volatility creates poor oxidation of the soot particle in the smoke, which increases the smoke emissions.^[^ ^42^, ^43^ ^]^ SGWPO75P25 has 1.35% and 16.59% reduced smoke emission compared to the diesel and SGWPO at full load. The pentanol addition in the SGWPO produced better evaporation and supplied oxygen content to combustion which increased the oxidation of the carbon soot particles in the fuel blend.^[^ ^33^, ^35^ ^]^

The SGWPO75P25+NG produced 10.23% and 24.09% smoke emissions lower than diesel and SGWPO, respectively. This reduction in smoke is because of the combined effect of both pentanol and graphene nanoparticles in the fuel. Pentanol produced better evaporation and additional oxygen content for better combustion. Nanographene produces catalytic engagement and reactivity engagement, which increase the carbon soot oxidation which reduces the smoke formation.^[^ ^31^, ^38^ ^]^ Similarly, SGWPO75P25+NSDO has 16.14% and 29.10% less smoke emission than the diesel and SGWPO, respectively. This reduction is because of both pentanol and the nano SiO_2_ combined effect involvement in the combustion. The enhanced evaporation, excess oxygen supply by the pentanol and the SiO_2_ produced a higher catalytic engagement, which converted a higher amount of the carbon soot into CO_2_ with the support of their oxidation capacity.^[^ ^30^ ^]^ Due to its high viscosity and low volatility, SGWPO prevents adequate atomization and vaporization, which leaves the air and fuel not fully mixed. Increased soot production and smoke emissions are caused by the incomplete oxidation of carbon particles during combustion. Due to its increased density and viscosity, which affects its spray and atomization properties, SGWPO emits 18.27% more smoke than diesel. SGWPO's low volatility slows down combustion, which results in less soot particle oxidation and higher smoke emissions. By increasing evaporation and decreasing viscosity, pentanol improves atomization and creates a more homogeneous air‐fuel mixture. When employing the SGWPO75P25 fuel blend, the higher oxygen level in pentanol helps to improve the oxidation of soot particles during combustion. There are less unburned carbon particles due to improved vaporization and oxygen availability, which lowers smoke emissions by 16.59% as compared to SGWPO. In addition to supporting a more controlled combustion process, pentanol's cooling impact helps to reduce soot.

During combustion, nanographene enhances the oxidation of carbon soot particles by acting as a catalyst when using the fuel blend of SGWPO75P25+NG. The blend's pentanol increases fuel atomization and adds more oxygen, which promotes more efficient burning. Nanographene's thermal engagement and catalytic capabilities speed up particle oxidation, lowering its concentration in the exhaust. As pentanol's improved vaporization and nanographene's catalytic action work together, smoke emissions are reduced by 24.09% as compared to SGWPO. By serving as a catalyst and supplying more oxygen for soot oxidation, SiO_2_ nanoparticles improve combustion. Pentanol and SiO_2_ nanoparticles work together to minimize incomplete combustion by creating a fuel‐air mixture that is evenly and thoroughly blended. While SiO_2_ promotes the catalytic oxidation of carbon soot into CO₂, which greatly lowers smoke emissions, pentanol enhances evaporation and provides oxygen. Because SiO_2_ nanoparticles have exceptional oxidation efficiency and catalytic effects, the SGWPO75P25+NSDO blend produces the lowest smoke emissions, reducing them by 29.10% when compared to SGWPO and 16.14% when compared to diesel. **Table** **8** demonstrates the comparative analysis of fuel blends’ performances in terms of smoke emissions and highlights the key factors influencing the results.

**Table 8:** Comparison of Fuel Performances at full load in terms of Smoke emissions.

Fuel blend	Smoke emission [m^3^ kg^−1^]	Change vs diesel [%]	Change vs SGWPO [%]	Key factors influencing smoke emission
Diesel	188.3	Baseline	−18.27%	Efficient combustion with optimal viscosity and volatility reduces smoke.
SGWPO	222.7	18.27%	Baseline	High viscosity and poor volatility hinder soot oxidation, increasing smoke.
SGWPO75P25	185.8	−1.35%	−16.59%	Pentanol improves evaporation and oxygen availability, enhancing soot oxidation.
SGWPO75P25+NG	169.1	−10.23%	−24.09%	Nanographene and pentanol enhance the oxidation of soot particles, reducing smoke.
SGWPO75P25+NSDO	157.9	−16.14%	−29.10%	SiO_2_ and pentanol improve catalytic engagement and oxidation capacity.

**Figure 9:**
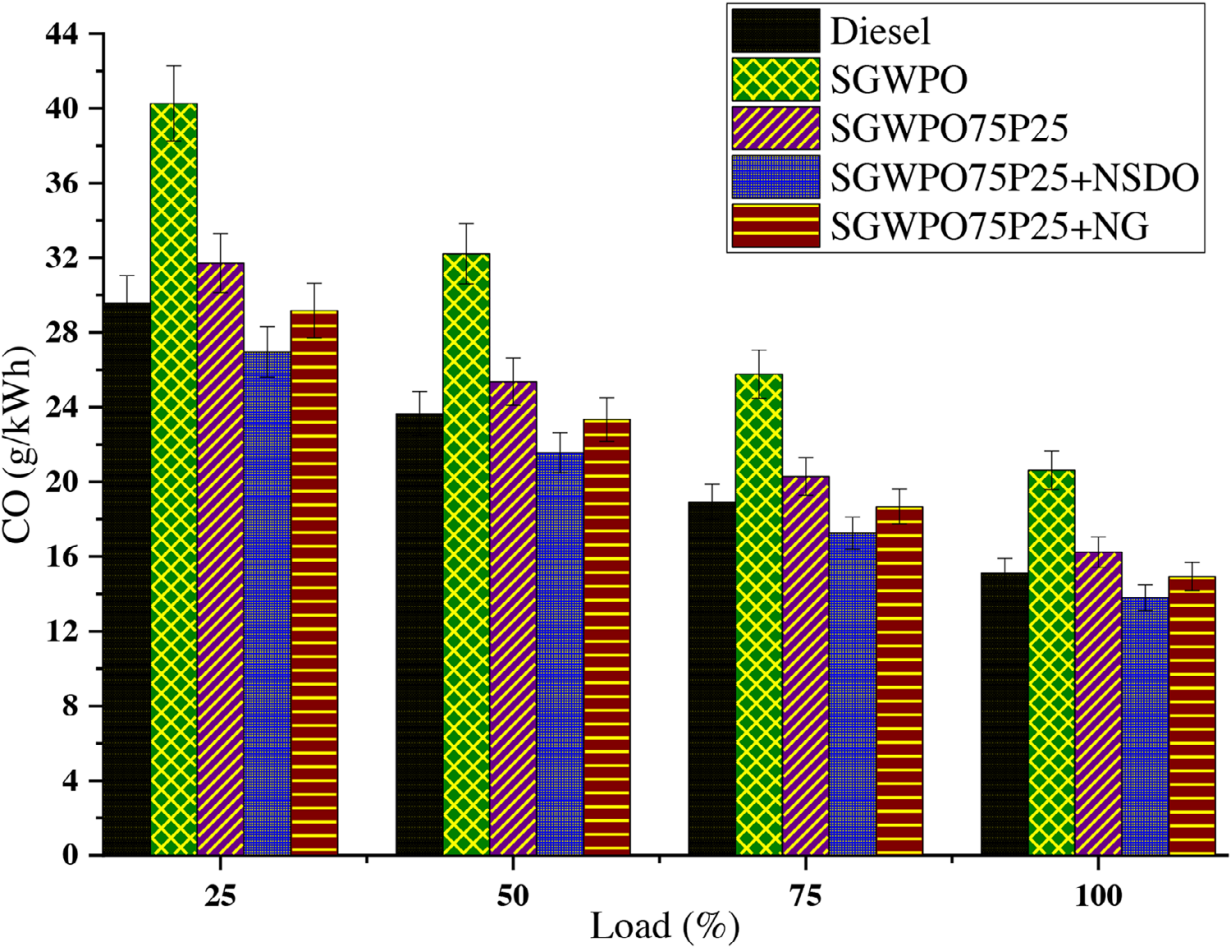
CO emission variation by blends and nano additives influences the SGWPO in the emission analysis.

Incomplete combustion, in which fuel is only partially oxidized because of inadequate oxygen or incorrect fuel‐air mixing, produces carbon monoxide. Carbon monoxide emissions variation by blends and nano additives influence on the SGWPO in the combustion analysis is shown in **Figure** **9** at different loads. Compared to a lower load, the higher load has lower CO emissions for all the test fuels. At maximum load, the highest CO emission of 20.62 g kWh^−1^ is produced by SGWPO, and the lowest CO emission of 13.80 g kWh^−1^ is obtained by SGWPO75P25+NSDO. Compared to diesel, SGWPO has 36.17% increased CO emission owing to incomplete combustion with higher viscosity and lag of oxygen availability in the fuel, increasing the CO emission.^[^ ^8^, ^18^ ^]^ SGWPO75P25 has 7.23% higher and 21.25% lower CO emissions than the diesel and SGWPO at full load. The pentanol addition in the SGWPO produced better evaporation, higher volatility and supplied oxygen content to combustion which increased the complete combustion and reduced the CO emission.^[^ ^20^, ^35^ ^]^ The SGWPO75P25+NG has produced 1.34% and 27.55% CO emissions less than diesel and SGWPO, respectively. This reduction in CO emission is because of the shared consequence of both pentanol and the nanoparticles of graphene in the fuel. Pentanol produced improved evaporation and supplementary oxygen content for better combustion. Nanographene forms the catalytic engagement and reactivity engagement which increase the CO oxidation into CO_2_, which reduces CO emission.^[^ ^38^ ^]^ The CO emission of SGWPO75P25+NSDO is lesser than SGWPO75P25+NG due to the carbon content available in the nanographene.

Similarly, SGWPO75P25+NSDO has 8.85% and 33.06% less CO emissions than the diesel and SGWPO, respectively. This reduction is because of both pentanol and the nano SiO_2_‐combined effect involvement in the combustion. The improved evaporation and excess oxygen supply by the pentanol and the SiO_2_ formed a higher catalytic engagement, which converted available CO emissions into carbon dioxide emissions, and the reactivity engagement created with the available oxygen content in the pentanol and SiO_2_ participated and converted the available CO further oxidation, resulting in reduced CO emissions.^[^ ^30^, ^39^ ^]^ Because of its high viscosity and low volatility, SGWPO is difficult to properly atomize and burn, which results in incomplete carbon oxidation to CO₂ and increased CO emissions. Due to the disruption of the atomization process caused by its higher viscosity, which results in larger fuel droplets and poor vaporization, SGWPO releases 36.17% more CO than diesel. The localized oxygen shortage caused by the delayed combustion encourages incomplete oxidation and the production of CO. However, oxygenated fuels could increase oxidation reactions by introducing more oxygen into the combustion process and improving fuel‐air mixing.^[^ ^44^ ^]^ Pentanol's increased volatility makes it possible for better vaporization and more effective combustion. Despite being 7.23% higher than diesel due to SGWPO's intrinsic combustion inefficiencies, pentanol's oxygen promotes full combustion, lowering CO emissions by 21.25%. More complete carbon oxidation into CO₂ is ensured by pentanol's contribution to the SGWPO75P25 fuel blend, which improves evaporation and flame stability.

By offering reactive spots for the reaction, graphene nanoparticles function as combustion catalysts, encouraging the oxidation of CO into CO₂. The blend's pentanol further improves oxygen availability and evaporation, which lowers the amount of CO produced during burning. Nanographene's catalytic activity speeds up combustion chamber oxidation processes, more efficiently reducing CO to CO₂. Pentanol's oxygen and nanographene's catalytic qualities work together in the SGWPO75P25+NG Blend to produce CO emissions that are 27.55% lower than those of SGWPO and even marginally lower than diesel because of improved oxidation efficiency. During combustion, SiO_2_ nanoparticles serve as a catalyst, promoting oxidation processes that change CO into CO₂. Pentanol increases oxygen availability and evaporation, which increases combustion efficiency. Compared to nanographene, SiO_2_ nanoparticles offer more catalytic engagement, guaranteeing a more complete conversion of CO to CO_2_. Pentanol's oxygen concentration enhances this effect, making SGWPO75P25+NSDO the most effective mix for reducing CO emissions, with emissions 33.06% lower than SGWPO and 8.85% lower than diesel. **Table** **9** demonstrates the comparative analysis of fuel blends’ performances in terms of CO emissions and highlights the key factors influencing the results.

**Table 9:** Comparison of Fuel Performances at full load conditions in terms of CO emissions.

Fuel blend	CO emissions [g kWh^−1^]	Change vs diesel [%]	Change vs SGWPO [%]	Key factors influencing CO emissions
Diesel	15.48	Baseline	−36.17%	Balanced combustion with moderate viscosity and oxygen content.
SGWPO	20.62	36.17%	Baseline	Incomplete combustion due to higher viscosity and inadequate oxygen supply.
SGWPO75P25	16.6	7.23%	−21.25%	Pentanol addition improves evaporation, volatility, and oxygen availability.
SGWPO75P25 +NG	15.27	−1.34%	−27.55%	Nanographene enhances CO oxidation to CO₂ through catalytic engagement.
SGWPO75P25 +NSDO	13.8	−8.85%	−33.06%	The combined effect of pentanol and nano‐SiO_2_ improves oxidation and combustion efficiency.

**Figure 10:**
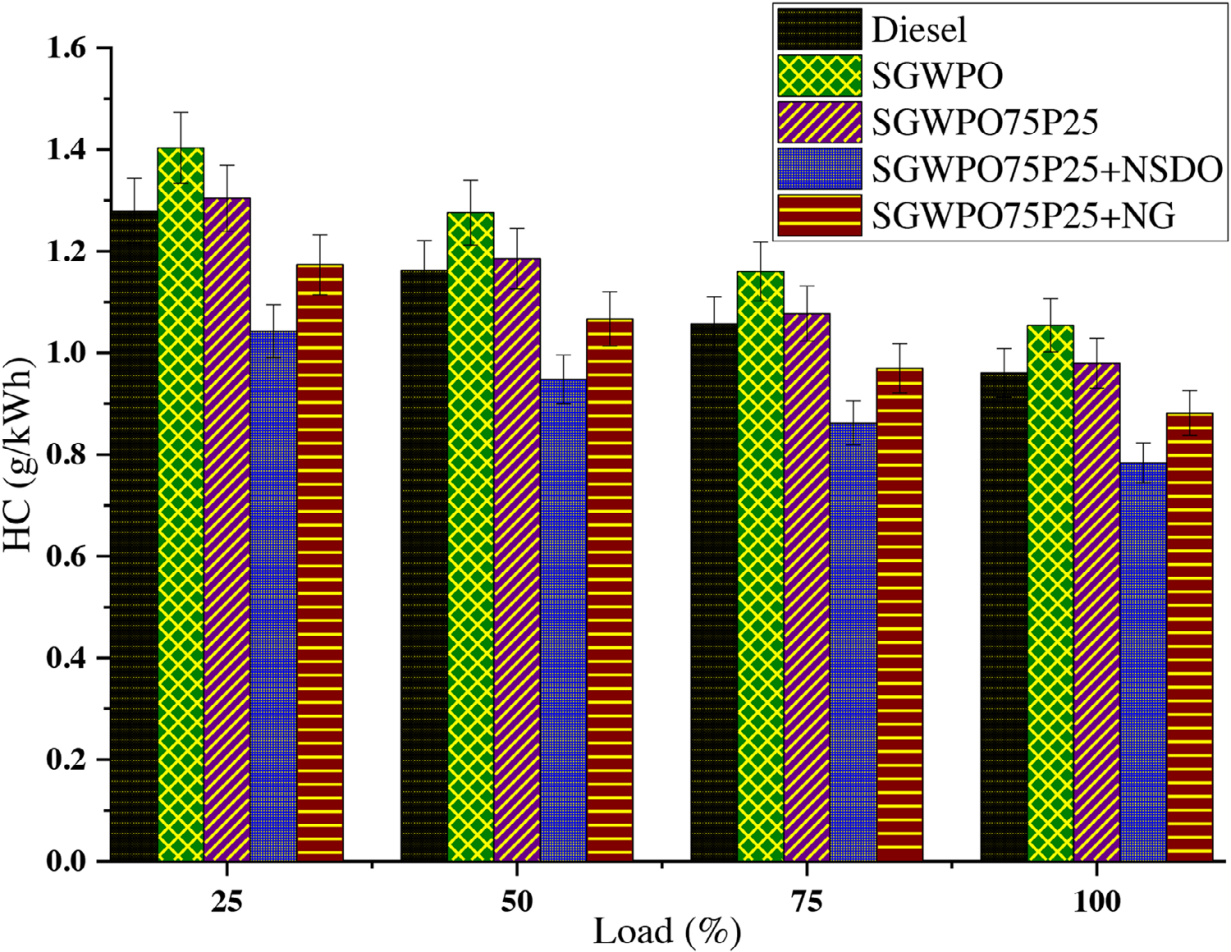
HC emission variation by blends and nano additives influences the SGWPO in the emission analysis.

When there is inadequate oxidation, inadequate fuel‐air mixing, or localized cooling, incomplete combustion results in HC emissions. Indeed, HC emissions variation by blends and nano additives influences the SGWPO in the combustion analysis, as mentioned in **Figure** **10** at different loads. Compared to a lower load, the higher load has lower HC emissions for all the test fuels. At maximum load, the highest HC emission of 1.054 g kWh^−1^ is produced by SGWPO, and the lowest HC emission of 0.784 g kWh^−1^ is obtained by SGWPO75P25+NSDO. Compared to diesel, SGWPO has a 9.72% increase in HC emission owing to higher viscosity, leading to delayed combustion, which increases the HC emission.^[^ ^8^, ^45^ ^]^ SGWPO75P25 has 1.94% higher and 7.09% lower HC emissions than the diesel and SGWPO at full load. The pentanol addition in the SGWPO produced better evaporation, higher volatility and supplied oxygen content for better combustion, which increased the complete combustion and reduced HC emissions.^[^ ^20^, ^33^ ^]^

The SGWPO75P25+NG has produced 8.25% and 16.38% less hydrocarbon emissions than diesel and SGWPO, respectively. This reduction in hydrocarbon emission is because of the shared consequence of both the pentanol and graphene nanoparticles in the fuel. Pentanol produced improved evaporation and supplementary oxygen content for better combustion. Nanographene formed the catalytic engagement and reactivity engagement which will enhance the combustion and reduce the hydrocarbon emission.^[^ ^31^, ^38^ ^]^ Due to the higher carbon content available in the nanographene, the hydrocarbon emission of SGWPO75P25+NG is higher than SGWPO75P25+NSDO. Similarly, SGWPO75P25+NSDO has 18.44% and 25.67% less hydrocarbon emission than the diesel and SGWPO, respectively, this reduction is because both pentanol and the nano‐SiO_2_ combined effect helped for a better combustion. The improved evaporation and excess oxygen supply by the pentanol and SiO_2_ formed the higher catalytic engagement and the reactivity engagement for better combustion and better oxidation with the available oxygen content, which reduced the hydrocarbon emission.^[^ ^30^, ^46^ ^]^ Because of its reduced volatility and increased viscosity, SGWPO prevents the fuel's hydrocarbon molecules from burning completely. Due to its higher viscosity, SGWPO has poorer atomization, which results in 9.72% more UHC emissions than diesel. decreased volatility, which results in partial mixing with air and delayed evaporation. Unburned fuel is released as hydrocarbons due to an insufficient oxygen supply. By adding more oxygen to the fuel blend and increasing volatility, pentanol improves the fuel's characteristics and promotes better combustion. This decreases unburned hydrocarbons and enhances flame propagation. Because of its increased volatility, pentanol evaporates more quickly, improving the fuel‐air mixture. Hydrocarbon molecules are oxidized with the help of pentanol's oxygen content. Although SGWPO's intrinsic combustion inefficiencies result in 1.94% higher UHC emissions than diesel, the SGWPO75P25 blend outperforms pure SGWPO by a wide margin.

As catalysts for combustion, nanographene nanoparticles speed up reactions and encourage full combustion. By increasing the fuel's volatility and oxygen content, pentanol also helps to lower UHC. UHC formation is decreased by nanographene's enhancement of hydrocarbon oxidation. Better fuel utilization is made possible by graphene nanoparticles, which increase the surface area for combustion reactions.

In comparison to SGWPO75P25+NSDO, the blend benefits from both the enhanced combustion characteristics of pentanol and the catalytic activity of nanographene; however, the reduction in UHC is somewhat constrained by graphene's higher carbon content. Unburned hydrocarbons are more readily converted to CO₂ and water by SiO_2_ nanoparticles, which function as excellent combustion catalysts. Pentanol's enhanced evaporation and oxygen content enhance this catalytic activity, resulting in the greatest decrease in UHC emissions. By actively participating in oxidation reactions, SiO_2_ nanoparticles transform UHC into CO₂. By improving fuel atomization and mixing, pentanol lowers the amount of unburned hydrocarbon emissions. SGWPO75P25+NSDO is the most effective blend in terms of lowering UHC emissions, with emissions 18.44% lower than diesel and 25.67% lower than SGWPO. **Table** **10** demonstrates the comparative analysis of fuel blends’ performances in terms of HC emissions and highlights the key factors influencing the results.

**Table 10:** Comparison of fuel performances at full load conditions in terms of HC emissions.

Fuel blend	UHC emissions [g kWh^−1^]	Change vs diesel [%]	Change vs SGWPO [%]	Key factors influencing UHC emissions
Diesel	0.96	Baseline	−9.72%	Balanced combustion with moderate viscosity and adequate oxygen content.
SGWPO	1.054	9.72%	Baseline	combustion delay due to higher viscosity and reduced volatility.
SGWPO75P25	0.941	1.94%	−7.09%	Pentanol improves evaporation, volatility, and oxygen availability.
SGWPO75P25+NG	0.881	−8.25%	−16.38%	Nanographene enhances combustion through catalytic activity and heat transfer.
SGWPO75P25+NSDO	0.784	−18.44%	−25.67%	The combined effect of pentanol and nano‐SiO_2_ improves oxidation and combustion efficiency.

### 4.3 Blends and Nano Additives Influence on the SGWPO in the Performance Analysis

**Figure 11:**
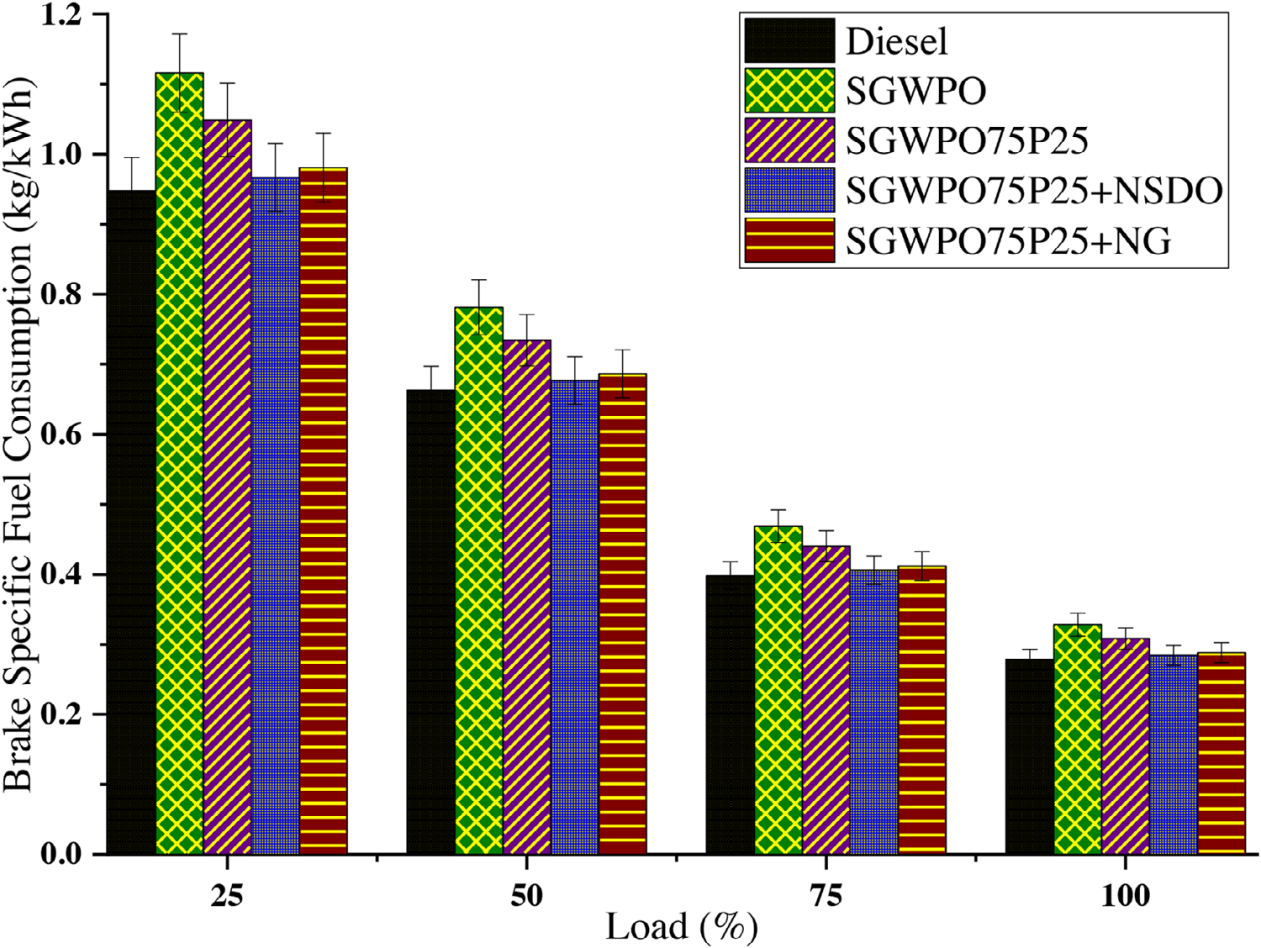
BSFC variation by blends and nano additives influence on the SGWPO in the performance analysis.

Brake‐specific fuel consumption (BSFC) calculates how much fuel is needed to generate one unit of power. A higher BSFC denotes less efficient fuel use, frequently as a result of less‐than‐ideal combustion. BSFC variation by blends and nano additives influence on the SGWPO during the combustion analysis is mentioned in **Figure** **11** at different loads.

At full load, the lowest BSFC of 0.279 kg kWh^−1^ is produced by diesel and the highest BSFC of 0.328 kg kWh^−1^ is obtained by SGWPO. Compared to diesel, SGWPO has 17.74% higher BSFC due to the lesser heating value of the fuel and poor vaporization formed through the higher viscosity of the SGWPO, requiring a higher amount of the fuel to produce the same power output.^[^ ^9^, ^41^ ^]^ The addition of pentanol improves the calorific value and vaporization quality, therefore, SGWPO75P25 has produced a 6.03% reduction in BSFC compared to the SGWPO. The further increased heating value by the addition of the nanographene, therefore, SGWPO75P25+NG has produced 3.45% higher and 12.14% lesser BSFC than diesel and SGWPO, respectively. This difference in the BSFC is because of the increased heating value of the nano‐fuel.^[^ ^31^ ^]^ Similarly, SGWPO75P25+NSDO has 1.99% higher and 13.38% lower BSFC than the diesel and SGWPO respectively, these variations in BSFC are because of the improved heating value of the nano fuel, the available oxygen content with pentanol and SiO_2_ with their better oxidation and increased micro explosion improves the combustion efficiency.^[^ ^47^, ^48^, ^49^ ^]^

SGWPO doesn't have as much calorific value as diesel, so more fuel is needed to produce the same amount of energy. Due to incomplete combustion caused by SGWPO's poor atomization and vaporization, fuel consumption rises and energy output falls. Compared to diesel, SGWPO has a 17.74% higher BSFC because it burns more fuel to make up for its inefficiencies. By improving fuel characteristics like volatility and heating value, pentanol enhances combustion, improves fuel‐air mixing, and uses less fuel. Fuel evaporation is improved by pentanol's lower boiling point, which improves atomization. Pentanol's oxygen content encourages full combustion, which uses less fuel. SGWPO75P25 has a 6.03% lower BSFC than SGWPO, which suggests more effective energy use. By increasing the fuel's energy density and serving as catalysts for combustion, nanographene nanoparticles speed up the oxidation of hydrocarbons and lower the amount of fuel that is needed. Enhanced heating value: By raising the blend's calorific value, nanographene produces more energy per unit of fuel. The high surface area of nanographene accelerates oxidation reactions and increases combustion efficiency. The BSFC of SGWPO75P25+NG is 12.14% lower than that of SGWPO. Due to SGWPO's lingering inefficiencies, its BSFC is still 3.45% higher than diesel's. Through their catalytic action, SiO_2_ nanoparticles improve combustion and fuel efficiency by promoting improved fuel oxidation and micro‐explosions. By making hydrocarbons more reactive, SiO_2_ nanoparticles guarantee more thorough combustion. Larger fuel droplets are broken up into smaller particles by the quick evaporation of SiO_2_’s water molecules, which improves combustion. The presence of oxygen in pentanol and SiO_2_’s catalytic function causes a 13.38% decrease in BSFC when compared to SGWPO. SGWPO75P25+NSDO has a 1.99% higher BSFC than diesel in spite of this, suggesting that there are still some minor inefficiencies when compared to fossil fuels. **Table** **11** demonstrates the comparative analysis of fuel blends’ performances in terms of BSFC and highlights the key factors influencing the results.

**Table 11:** Comparison of fuel performances at full load conditions in terms of BSFC.

Fuel blend	BSFC [kg kWh^−1^]	Change vs diesel [%]	Change vs SGWPO [%]	Key factors influencing BSFC
Diesel	0.279	Baseline	−17.74%	High heating value and good vaporization lead to efficient power output.
SGWPO	0.328	17.74%	Baseline	Poor vaporization and higher viscosity require more fuel for the same power output.
SGWPO75P25	0.308	10.39%	−6.03%	Pentanol enhances calorific value and reduces viscosity, improving combustion.
SGWPO75P25 +NG	0.289	3.45%	−12.14%	Nanographene increases heating value, improving combustion efficiency.
SGWPO75P25 +NSDO	0.285	1.99%	−13.38%	Pentanol and SiO_2_ improve oxidation, micro‐explosions, and combustion.

**Figure 12:**
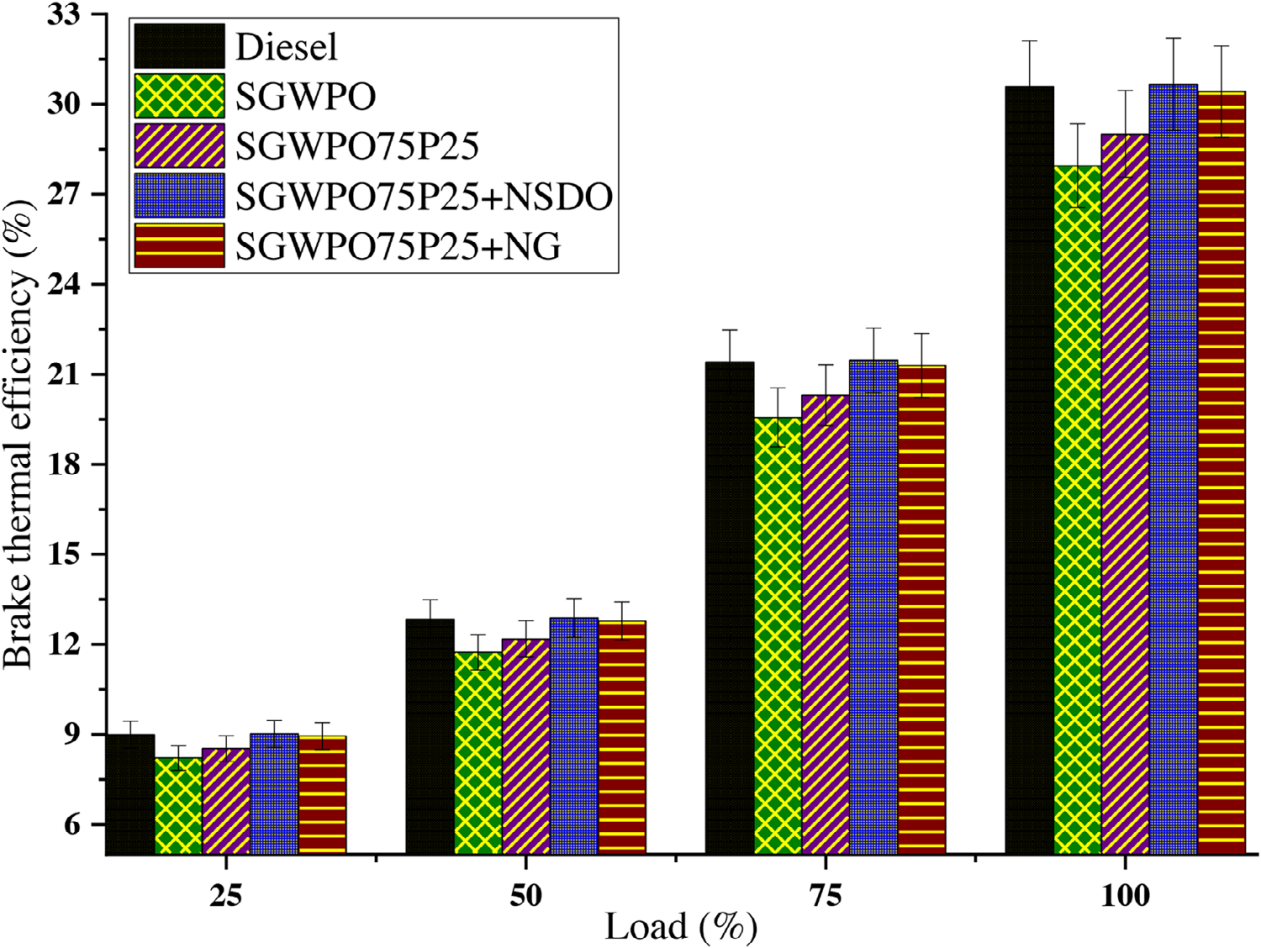
BTE variation by blends and nano additives influences the SGWPO in the performance analysis.

Brake thermal efficiency (BTE) calculates how well the engine transforms the energy in the fuel into work that is useful. Suboptimal combustion and energy use are indicated by lower BTE. The BTE variation by blends and nano additives influences the SGWPO in the combustion analysis, is mentioned in **Figure** **12** at different loads. At full load, the lowest BTE of 27.95% is produced by SGWPO and the highest BTE of 30.67% is achieved by SGWPO75P25+NSDO. As per Equation (3), the increased BSFC simultaneously reduced the BTE. Compared to diesel, SGWPO has 8.60% less BTE due to the higher fuel consumption because of their lesser heating value, higher viscosity and combustion delay.^[^ ^18^, ^50^ ^]^

The addition of pentanol improves the vaporization quality and heating value; therefore, SGWPO75P25 has produced a reduced BSFC, which increased by 3.77% BTE compared to the SGWPO due to better combustion.^[^ ^35^ ^]^ The nanographene further increased the heating value of the blend, therefore, SGWPO75P25+NG produced 0.53% less and 8.83% higher BTE than diesel and SGWPO, respectively. This difference in the BTE is because of the increased heating value and better combustion with higher HRR.^[^ ^27^, ^38^ ^]^ Similarly, SGWPO75P25+NSDO has 0.28% and 9.72% higher BTE than the diesel and SGWPO respectively, this discrepancies in BTE is because of the enhanced heating value of the nano fuel with nano‐SiO_2_, better combustion with available oxygen content with pentanol and SiO_2_, and their better oxidation with increased micro explosion further improve the combustion with higher HRR.^[^ ^23^, ^35^ ^]^

Compared to diesel, SGWPO has a lower calorific value, which means that less energy is converted. SGWPO's higher viscosity causes energy losses because poor atomization and vaporization prevent full combustion. BTE decreases when more fuel is used to achieve the same result. Because of its higher ignition point and lower heating value, SGWPO has a 8.60% lower BTE than diesel. Pentanol increases thermal efficiency by improving vaporization and heating value, which improves combustion. The lower boiling point of pentanol promotes faster evaporation and finer atomization, which improves fuel‐air mixing efficiency. More oxygen is provided by pentanol, which encourages full combustion and lowers energy losses. Because of these factors (which include lower BSFC and improved energy conversion), the BTE of the SGWPO75P25 Blend is 3.77% higher than that of SGWPO. **Table** **12** demonstrates the comparative analysis of fuel blends’ performances in terms of BTE and highlights the key factors influencing the results.

**Table 12:** Comparison of fuel performances at full load conditions in terms of BTE.

Fuel blend	BTE [%]	Change vs diesel [%]	Change vs SGWPO [%]	Key factors influencing BTE
Diesel	30.59	Baseline	8.60%	High heating value and efficient combustion result in optimal BTE.
SGWPO	27.95	−8.60%	Baseline	Higher fuel consumption due to lower heating value, higher viscosity, and delayed combustion reduces BTE.
SGWPO75P25	28.99	−5.24%	3.77%	Pentanol addition improves vaporization, heating value, and combustion efficiency.
SGWPO75P25+NG	30.43	−0.53%	8.83%	Nanographene increases heating value and combustion rate, enhancing HRR and BTE.
SGWPO75P25+NSDO	30.67	0.28%	9.72%	Nano‐SiO_2_ and pentanol improve heating value, oxygen availability, and oxidation, boosting BTE.

Graphene nanoparticles improve the energy density and combustion quality of fuel by acting as catalysts for combustion. Because nanographene increases the blend's calorific value, more energy is produced per unit of fuel. By increasing oxidation rates and combustion kinetics, nanographene improves energy use and heat release.^[^ ^51^, ^52^ ^]^ The blend's rapid and effective heat release increases thermal efficiency, showing that the BTE from SGWPO75P25+NG blend is 8.83% higher than that of SGWPO, suggesting minor residual inefficiencies. Through catalytic effects and the micro‐explosion phenomenon, SiO_2_ nanoparticles improve combustion even more, maximizing fuel efficiency. By catalyzing combustion reactions, SiO_2_ nanoparticles guarantee more thorough fuel oxidation. Larger fuel droplets are broken up by the quick evaporation of water molecules inside the nanoparticles, which increases the surface area and efficiency of combustion. The oxygen content of pentanol and the catalytic activity of SiO_2_ combine to produce effective fuel oxidation and increased heat release. SGWPO75P25+NSDO exhibits superior thermal efficiency with the highest BTE of 30.67%, 9.72% higher than SGWPO and 0.28% higher than diesel. **Table** **13** compares the obtained results in this study with others.

**Table 13:** Comparative analysis.

Waste source	Study focus	Findings	Refs.
Low‐density polyethylene (LDPE) plastic waste	Combustion, emission, and performance evaluation of pyrolyzed oil from LDPE waste using exhaust gas recirculation at 0%, 10%, and 20% in a 5.2 kW engine.	At 20% exhaust gas recirculation, pyrolyzed oil achieved a BTE of 31.63%, along with a 62.28% reduction in NOx emissions (596 ppm) compared to diesel at full load.	[9]
Single‐use LDPE plastic waste	Investigated dual‐fuel operation with waste plastic oil and varying hydrogen flow rates (3–12 lpm).	With 12 lpm hydrogen, there was a 22.89% increase in BTE, a 15.88% rise in peak pressure, and a 60.85% improvement in heat release rate. Emission reductions included 53.42% lower smoke, 48.94% less CO₂, 53.99% less HC, and 86.86% less CO.	[18]
Waste plastic oil with 1‐pentanol	Evaluated blends of 10%, 20%, and 30% 1‐pentanol with waste plastic oil at 2000 rpm and varying engine loads (20–80%) under 10% and 20% exhaust gas recirculation.	The 30% 1‐pentanol blend exhibited a 3.3% drop in efficiency, a 0.02 kg kWh^−1^ increase in BSFC, 74 ppm lower NOx emissions, and 2.3 ppm higher HC emissions compared to diesel.	[35]
Waste plastic oil	Examined variations in exhaust gas recirculation, injection timings, and alcohol blends optimized using response surface methodology.	The WPO70P30 blend injected at 21°bTDC with 10% exhaust gas recirculation yielded optimal performance, with a desirability score of 0.968. Smoke emissions reduced by 76.8%, NOx emissions increased by 32%, and BSFC improved by 17.8%. Compared to neat WPO, smoke emissions decreased by 74.2%, NOx emissions rose by 9.7%, and BSFC improved by 3.2%.	[33]
High‐density polyethylene (HDPE) grocery bags	Assessed performance and emission characteristics for blends of up to 15% PPO with diesel fuel.	The blend with 5% PPO showed a BTE of 51.6%, compared to 47.44% for diesel. This indicates potential diesel savings of up to 5%.	[53]
Plastic waste and waste cooking oil	Evaluated ternary fuel blends containing 20% waste cooking oil biodiesel, 20% waste plastic oil, and 60% diesel.	Achieved a 1.71% improvement in BTE at 80% engine load. Emission reductions were observed for HC, CO, and NOx compared to pure diesel.	[54]
Surgical glove waste	Investigated pyrolysis oil blended with 75% pentanol (25% by volume) and infused with 100 ppm SiO_2_ nanoparticles or 100 ppm nanographene.	The nano‐SiO_2_ blend demonstrated superior performance, including a 0.81% improvement in BTE, 7.8% higher heat release rate, and emissions reductions of 8.25% (NOx), 7.51% (CO), 8.88% (smoke), and 10.91% (HC). It was identified as a better alternative compared to nanographene and neat pyrolysis oil.	This study

In general, these nano‐fuel‐based environmental and human effects are much less compared to other nanoparticles because of their lesser contribution in the combustion, which acts as a catalyst for improved combustion emission reduction, not as the main contributor. SiO_2_ is not a major pollutant in the environment because its toxicity is very low, and the carbon content is a negligible amount. It also reduces CO, HC, and smoke emissions compared to NG. This variation is because of the higher carbon content availability in the NG than in the NSDO.

## 5 Conclusion 

This study demonstrates the effective utilization of surgical glove waste as a sustainable fuel source for a 5.2‐kW CI engine through pyrolysis. Steam‐sterilized surgical glove waste pyrolysis oil (SGWPO) shows potential as an alternative fuel, albeit with lower performance and higher emissions compared to diesel at full load. Blending SGWPO with pentanol and further enhancing it with nanographene and nano‐SiO_2_significantly improved engine performance and reduced emissions.

Among the additives, nano‐SiO_2_ delivered superior results, with 9.72% higher BTE and substantial reductions in NOx (36.81%), smoke (29.1%), CO (33.06%), and hydrocarbon (25.67%) emissions compared to neat SGWPO. These findings emphasize the viability of nano‐SiO_2_‐enhanced SGWPO and pentanol fuel as an alternative, enabling improved engine performance without modifications and addressing waste management challenges.

From an economic analysis point of view, nano‐SiO_2_‐enhanced SGWPO has 7% and 18% higher values than the neat SGWPO and diesel, respectively. In the future, mass production of this SGWPO will reduce the cost of the fuel and also reduce the amount of surgical glove waste in the environment. Therefore, it supports the reduction of surgical glove waste and new alternate fuel for the CI engine with higher performance and lower emissions than diesel.

## 6 Future Scope

This study is limited to a specific engine configuration and controlled experimental conditions, which may not fully represent real‐world scenarios. The absence of exhaust gas recirculation (EGR) and advanced emission control techniques limits the scope of emission management evaluations. Future research should explore diverse operating conditions, such as varying injection pressures and timings, to optimize the straight use of SGWPO. Advanced combustion technologies like low‐temperature combustion, dual‐fuel operation, and reactivity‐controlled compression ignition could be investigated to further enhance performance and minimize emissions. Comprehensive life cycle assessments of SGWPO‐derived fuels are also essential to ensure their environmental and economic sustainability. Furthermore, scaling the production of SGWPO and evaluating its long‐term engine impacts would facilitate its transition from laboratory investigations to real‐world applications. Future studies should investigate the scalability of the proposed fuel for diverse engine sizes and applications. Exploring hybrid additives, alternative nanoparticle formulations, and integration with renewable fuels could unlock new avenues for enhancing fuel efficiency and sustainability. The adoption of machine learning models for performance optimization and predictive analysis could further advance the application of SGWPO as a competitive alternative fuel. Also, the syngas produced during SGWPO preparation needs to be studied with the CI engine as a gaseous fuel. An extensive study of this syngas will be conducted with a comparative analysis of the present gaseous fuels. With respect to the nanoparticles' participation, the impact on engine durability (e.g., nozzle wear and carbon deposition) needs to be further studied.

## Conflict of Interest

The authors declare no conflict of interest.

## Data Availability

The data that support the findings of this study are available from the corresponding author upon reasonable request.
